# TRAF2/3 deficient B cells resist DNA damage-induced apoptosis via NF-κB2/XIAP/cIAP2 axis and IAP antagonist sensitizes mutant lymphomas to chemotherapeutic drugs

**DOI:** 10.1038/s41419-023-06122-2

**Published:** 2023-09-08

**Authors:** Monika Vashisht, Huaibin Ge, Jessy John, Harlie A. McKelvey, Jingxin Chen, Zhangguo Chen, Jing H. Wang

**Affiliations:** 1 UPMC Hillman Cancer Center, Division of Hematology and Oncology, Department of Medicine, University of Pittsburgh, Pittsburgh, PA 15213 USA; 2 Department of Immunology, University of Pittsburgh, Pittsburgh, PA 15213 USA

**Keywords:** Immune cell death, B-cell lymphoma

## Abstract

Deletion of *TRAF2* or *TRAF3* in B cells prolongs their survival. However, it remains unknown whether deletion of such factors affects B cells’ ability to tolerate DNA damage, which can be induced by chemotherapeutics and cause apoptosis. Genetic alterations of *TRAF2* or *TRAF3* are observed in subsets of human B-cell lymphomas and B cell-specific deletion of *TRAF3* led to lymphoma development in aged mice. However, it remains unknown whether double deficiency of *TRAF2* and *TRAF3* accelerates B-cell lymphomagenesis. Here, we showed that B cell-specific TRAF2/3 double deficient (B-TRAF2/3-DKO) B cells were remarkably more resistant to DNA damage-induced apoptosis via upregulating cIAP2 and XIAP, which in turn attenuates caspase-3 activation. Mechanistically, resistance to DNA damage-induced apoptosis required NF-κB2, which effects by upregulating XIAP and cIAP2 transcription. B-TRAF2/3-DKO mice exhibited a shorter lifespan and succumbed to splenomegaly and lymphadenopathy. Unexpectedly, the incidence of B-cell lymphoma development in B-TRAF2/3-DKO mice was relatively rare (∼10%). Sequencing B cell receptor repertoire of diseased B cells revealed that TRAF2/3 deficiency caused abnormal oligoclonal or clonal expansion of B cells. While a fraction of mutant B cells (25–43%) from aged diseased mice harbored recurrent chromosomal translocations, primary B cells isolated from young B-TRAF2/3-DKO mice had no detectable chromosomal alterations, suggesting that TRAF2/3 deficiency per se does not cause evident genomic instability in B cells. Chemo-resistant TRAF3-deficient B-cell lymphomas were sensitized to chemotherapeutic drugs by blocking IAP activity using IAP antagonist. We conclude that double deficiency of *TRAF2* and *TRAF3* does not accelerate B-cell lymphomagenesis. Our studies provide insight into mechanisms regulating DNA damage-induced apoptosis and may help develop effective therapies targeting mutant B-cell lymphomas using IAP antagonist.

## Background

Non-Hodgkin’s lymphomas (NHL) are the fifth most common cancers in the US, and more than 90% of NHL are of B cell origin [1]. TRAF3 is a signaling adaptor of TNF receptor (TNFR) superfamily (e.g., CD40), toll-like receptor (TLRs), and others [2–4]. Recent studies also show that TRAF3 can serve as a checkpoint that inhibits B cell receptor (BCR) signaling [5, 6]. TRAF3 is expressed ubiquitously in various cell types and acts as a suppressor of NF-κB activation [2–4]. Germline TRAF3 deficiency causes postnatal lethality, and mutant mice die by 10 days of age [7]. TRAF2 is also a signaling adaptor functioning downstream of TNFRs (e.g., CD40). In resting B cells, TRAF3 associates with NF-κB inducing kinase (NIK) (a.k.a. MAP3K14), while TRAF2 associates with cIAP1 and cIAP2. Within this cytoplasmic complex, TRAF2 and TRAF3 heteromeric interaction allows cIAP1/2 to induce NIK degradation. In activated B cells, TRAF2 and TRAF3 are recruited to membrane rafts and bind to upstream receptors (e.g., CD40), which leads to TRAF3 degradation [8, 9], thereby releasing NIK. Alternatively, NIK may be released by TRAF3 sequestration achieved by direct interaction of LMP-1 with TRAF3 [10] or by interaction of MYD88 with TRAF3 [11]. NIK in turn phosphorylates inhibitory κB (IκB) kinase-α (IKKα), which then phosphorylates NF-κB2 p100. This phosphorylation induces degradation of the ankyrin repeat domain of NF-κB2 p100 and releases NF-κB2 p52. Released NF-κB2 p52 forms active NF-κB2 heterodimer with Rel-B or c-Rel.

Human mature B cell lymphomas, including multiple myeloma (MM), mantle cell lymphomas (MCL) or marginal zone lymphomas (MZL), frequently harbor mutations or deletions of TRAF2, TRAF3 or BIRC3 (a.k.a. cIAP2) [12–20]. For instance, 15% of MCL patients harbor mutations in TRAF2 or BIRC3 [20]. Genetic lesions of NF-κB pathways occurred in >35% of splenic MZL patients [16], including BIRC3 (11%), MAP3K14 (8%), and TRAF3 (10%) [16]. One study identified biallelic inactivation of TRAF3 (9/41, 22%) in B-cell lymphomas (CLL, MCL and MZL) with interstitial del(14)(q24.1q32.33) [19]. Hence, the TRAF2/TRAF3/BIRC3 pathway is frequently mutated in MCL and MZL. TRAF3 mutations in human patients also led to autoimmunity and increased risk of B cell malignancy [21]. However, the mechanisms by which this pathway leads to B cell lymphomagenesis remain incompletely understood. A better understanding of such pathological mechanisms may improve therapeutic strategies by uncovering new targets or exploiting previously unidentified vulnerability in the mutant lymphomas.

When TRAF3 is deleted specifically in B cells via CD19Cre (B-TRAF3-KO), mice develop autoimmune manifestations (e.g., splenomegaly, lymphocyte infiltration in liver and immune-complex deposition in kidney) at the age of 9–12 months [22]. *Traf3* deletion in mouse B cells predisposed B-TRAF3-KO mice to B cell lymphomagenesis at the age of >18 months [23]. B-TRAF3-KO B cells proliferated better than control B cells upon anti-IgM/IL-4 stimulation in vitro [5]. B-TRAF3-KO B cells also exhibited a higher level of NF-κB2 activation [5, 22, 24]. However, it remains incompletely understood how TRAF3 deficiency leads to B cell lymphomagenesis and whether TRAF3 deficiency can directly cause genomic instability in B cells. B-TRAF2-KO mice exhibit similar phenotypes to B-TRAF3-KO mice in B cell development and survival as well as lymph organ homeostasis [22, 24, 25]. TRAF2 deficiency in B cells also resulted in elevated NF-κB2 activation [24]; however, TRAF2 and TRAF3 may play opposite roles in regulating NF-κB1 activation [26, 27] that is important for human B cell lymphoma survival and proliferation. Hence, it is critical to address if TRAF2 and TRAF3 deficiency cooperates to enhance the frequency or accelerate the development of B cell lymphomagenesis. Further elucidating the role of TRAF2 and TRAF3 deficiency in B cell lymphomagenesis may help sensitizing these mutant lymphomas to chemotherapeutic drugs or other therapeutics.

Since B cell-specific *TRAF3* and/or *TRAF2* deficiency increases active NF-κB2, presumably active NF-κB2 might promote B cell survival. In this regard, TRAF3 deficiency also increased the level of active NF-κB2 in T cells and macrophages, which did not confer survival advantage to these cells [28, 29]. It remains unresolved to what extent active NF-κB2 can enhance B cell survival in B-TRAF2/3-DKO B cells upon treatment of chemotherapeutic drugs, and what mechanisms active NF-κB2 employs to promote B cell survival. Lastly, it remains unknown whether B-TRAF2/3-DKO B cells survive better in response to chemotherapeutic drugs and whether NF-κB2 is required for protecting B cells against chemotherapy-induced cell death.

In the current study, we showed that B-TRAF2/3-DKO B cells were more resistant to chemo-induced apoptosis and their resistance relied on NF-κB2 which is required for XIAP and cIAP2 transcription. We found that B-TRAF2/3-DKO mice developed B-cell lymphomas with a lower frequency than what previously reported in B-TRAF3-KO mice [23]. We also sequenced the BCR repertoire of diseased B cells and examined chromosomal alterations in mutant B cells. IAP antagonists sensitized chemo-resistant B-cell lymphomas to chemotherapeutic drugs in the absence of TRAF3. Our studies provide insight into mechanisms regulating DNA damage-induced apoptosis in B cells and may help develop effective therapies for TRAF2/3 mutant B-cell lymphomas using IAP antagonist.

## Methods

### Mouse models

*TRAF2*^flox/flox^ [25] and *TRAF3*^flox/flox^ mice were generated previously [22]. Wild type C57BL/6 (B6), and *NFκB2*^flox/flox^ mice were purchased from Jackson Laboratory. B cell-specific TRAF3 knockout (KO) mice were generated by crossing *TRAF3*^flox/flox^ allele with CD19Cre, referred to as B-TRAF3-KO throughout the study. *TRAF3*^flox/flox^ mice were employed as littermate control (LMC) for all experiments and we acknowledge the caveat of lacking a CD19Cre only control in some experiments. B cell-specific TRAF2 KO mice were generated by crossing *TRAF2*^*flox/flox*^ allele with CD19Cre, referred to as B-TRAF2-KO. B-TRAF2/TRAF3-DKO mice were generated by crossing CD19Cre-*TRAF3*^flox/flox^ mice with *TRAF2*^flox/flox^ mice. B cell-specific TRAF3 and NF-κB2 double KO (B-TRAF3/NF-κB2-DKO) mice were generated by crossing *NF-κB2*^flox/flox^ with CD19Cre-*TRAF3*^flox/flox^ mice. Six to 12 weeks old mice were used for most experiments. For survival study, we observed mice up to 24 months or until mice were morbid and euthanized according to institutional guidelines. BALB/c/Rag2^–/–^IL2Rγ^–/–^SirpaNOD (BRGS) mice were provided by mouse core facility of University of Colorado Anschutz Medical Campus (Aurora, CO). NOD-scid/IL2Rγ^–/–^ (NSG) mice were purchased from Jackson lab. To test the tumorigenicity of diseased B cells collected from spleen or ascites, purified B cells (10 × 10^6^) in 200μl medium were subcutaneously injected at the flank of BRGS or NSG mice (6−8 weeks). Mice were monitored up to 5 months for tumor growth. For animal studies, no randomization or blinding was used. Animal work was approved by the Institutional Animal Care and Use Committee of the University of Colorado Anschutz Medical Campus (Aurora, CO) and University of Pittsburgh Hillman Cancer Center.

### Antibodies (Abs), chemicals and chemical treatment

All Abs used in the study including for flow, immunofluorescence, and western blotting are listed in Supplementary Table 1. Chemicals were purchased from the following companies: Cytarabine (Ara-C) from Accela Chem Bio (Cas 147-94-4), Doxorubicin (Dox) (S1208), AZD5582 (AZD) (S7362), and SM-164 (SM) (S7089) from SelleckChem. All chemicals were dissolved in H_2_O or DMSO. When DMSO is diluted by 1:1000 or more with medium, there was no detectable effect on our results. We thus prepared chemicals at least 2000× stocks if dissolved by DMSO. Hence, we did not set up DMSO control in all experiments. Lipopolysaccharides (LPS) (E. coli 0111: B4) was purchased from Sigma. Mouse IL-4 (Z02996) was purchased from GenScript Biotech.

### Cell culture and flow cytometry

OCI-Ly1 (Ly1) and OCI-Ly7 (Ly7) human lymphoma cell lines were described previously [30]. OCI-LY1 and OCI-LY7 were obtained from Dr. Wing C. (John) Chan (City of Hope Medical Center, Duarte, CA) and cultured as described previously [30]. The cell line authentication and Mycoplasma testing were performed by Molecular Biology Service Center at the Barbara Davis Center (University of Colorado, Anschutz Medical Campus, Aurora, CO) in 2019. The cells were tested and reauthenticated by PCR assays as described (http://www.barbaradaviscenter.org/). Ly1 cells express TRAF2 and TRAF3 protein, while TRAF3 protein is undetectable in Ly7 cells designated as TRAF3-loss cell line. TRAF3 or TRAF2 was deleted in Ly1 cells using TRAF3 CRISPR/Cas9 KO plasmid (h, sc-400473-KO-2, SCBT) or TRAF2 CRISPR/Cas9 KO plasmid (h, sc-400361, SCBT), respectively, with 4D-Nucleofector system (Lonza). TRAF2/3 double deletion in Ly1 cells was achieved by deleting TRAF3 then TRAF2 sequentially using CRISPR/Cas 9 KO plasmids described above. Deletion was confirmed by western blotting with TRAF2 and TRAF3 specific antibodies. Lymphoma cells were cultured in RPMI1640 medium supplemented with 10% fetal bovine serum (FBS), 100μM of β2-mercaptoethanol (M3148-100ML, Sigma), 1% of antibiotics (15240-062, Gibco), L-glutamine (25030-081, Gibco), MEM non-essential amino acid (25-025-CI, Corning), HEPES (25-060-CI, Corning) and sodium pyruvate (11360-070, Gibco). For apoptosis assay, lymphoma cells were treated with Ara-C, DOX or AZD at the indicated concentrations either alone or in combination for 18 h, then harvested and stained by APC-conjugated Annexin-V (550474, BD Pharmingen) and Aqua (L34966A, Invitrogen) according to Annexin-V staining protocol. Primary splenic B cells were purified using EasySep mouse B cell isolation kit (19854 A, STEMCELL Technologies) according to manufacturer’s instructions. For survival or apoptosis assay, purified splenic B cells (1 × 10^6^/ml, 1 ml/well in 24-well plate) were cultured in lymphocyte medium described above for 16 h in the presence or absence of Ara-C (10 μg/ml) with or without other chemicals as indicated in individual experiments. Cells were harvested, stained, and analyzed by flow cytometry. For kinetic apoptosis assay, purified splenic B cells were cultured as described above. NucView® caspase-3 substrates (Nuc3) contain novel fluorogenic DNA dyes coupled to the caspase-3/7 recognition sequence (DEVD). Nuc3 is initially non-fluorescent. During apoptosis, caspase-3/7 cleaves the substrate and releases the high-affinity DNA dye leading to nuclear fluorescent staining. NucView® 488 Caspase-3 Substrate (10402, Biotium) was added into cell culture with or without Ara-C (10 μg/ml) for 0, 3, 6, 9, 12 and 15 h followed by Aqua staining. Flow cytometry was performed on Beckman Coulter CytoFlex 6 L or BD LSRFortessa. Data were analyzed with Flow-Jo software. To establish lymphoma cell lines, purified B cells were cultured in lymphocyte medium described above or StemSpan SFEM II medium (09655, STEMCELL) supplemented with 10% FBS and antibiotics in presence or absence of mouse IL-4 or BAFF (8876-BF-010/CF, R&D system). Medium was replenished every 3–5 days. Cells were observed up to 20 weeks. Cells that proliferated and expanded during the time were defined as cell lines.

### Immunofluorescence

Purified splenic B cells were seeded in 6-well plates (1 × 10^6^ cells/ml, 3 ml/well) in lymphocyte medium. NucView® 488 Caspase-3 Substrate was added with Ara-C (10 μg/ml) for 16 h. Coverslips were treated with 0.01% poly-L-lysine (P4707-50 ml, Sigma-Aldrich). Cells were collected and washed twice with 1×PBS and loaded onto the coverslips in duplicate. After 60-minute incubation, coverslips were washed with 1×PBS twice. Cells were fixed with 4% paraformaldehyde for 30 min at room temperature (RT) followed by washing with 1×PBS thrice. Cells were permeabilized with 0.5% Triton-X-100 for 15 min at RT followed by washing with 1×PBS thrice. Cells were blocked with blocking buffer (1×PBS containing 3% BSA and 0.2% Tween 20) 1 h at RT. Coverslips were rinsed with blocking buffer once, incubated up-side-down with anti-γ-H2AX antibody overnight at 4 °C followed by Alexa Fluor® 647-conjugated goat anti-rabbit secondary antibody for 1 h at RT. After incubation, coverslips were rinsed with blocking buffer 7 times. Mounting medium containing DAPI was dropped on slides and coverslips were placed up-side-down on Vecta shield mounting medium (Cat. H-1200, Vector Laboratories). Coverslips were sealed and images were acquired with an Eclipse TE2000 (Nikon).

### Biochemical assays and Western blotting

For detecting apoptosis-related protein expression or activation in Ara-C treated LMC or B-TRAF2/3-DKO B cells, we employed proteome profiler mouse apoptosis array kit (ARY031, R&D system) according to manufacturer’s instructions. Briefly, purified splenic B cells of LMC and B-TRAF2/3-DKO mice were treated with Ara-C in RPMI1640 lymphocyte medium for 16 h. Cells were harvested and lysed with lysis buffer provided by the kit. Sample loading and target protein detection were performed according to kit protocol. Relative expression level of proteins of interest was evaluated by dot density with ImageJ/Fiji system.

The expression level of individual proteins was validated by western blotting as described. LMC and B-TRAF2/3-DKO B cells were treated as above, harvested and lysed with lysis buffer (50 mM Tris-base pH7.5, 150 mM NaCl, 1 mM EDTA, 2 mM Na_3_O_4_V, 4 mM NaF, 1% Triton-X100, 0.1% SDS, 0.5% Sodium deoxycholate) for 30 min on ice. Lysates were centrifuged at 12000 RPM for 10 min at 4 °C. Supernatants were collected for subsequent analysis. Protein concentrations were determined with Pierce BCA protein assay kit (23228, Thermo scientific). Total protein lysate (35μg) per sample was separated on SDS-PAGE and transferred onto 0.2 μm nitrocellulose membranes (1620112, Bio-Rad). Membranes were blocked with 6% dried milk in wash buffer (0.1% Tween 20 TBS buffer) and probed with specific Abs followed by HRP-conjugated anti-mouse or rabbit secondary Abs, respectively. Protein bands were read with ECL Plus (Cytiva) on a G:Box Chemi-XX6 platform (Syngene, Frederick, MD).

### Chromatin immunoprecipitation (ChIP)

To prepare chromatin samples, purified splenic B cells (30 × 10^6^) of LMC or B-TRAF2/3-DKO mice were cultured in lymphocyte media (15 ml) and treated with Ara-C (20μg/ml) for 16 h. Cells were harvested and chromatin was cross-linked by adding 0.405 ml of 37% formaldehyde (F79-500, Fisher Scientific, Fairlawn, NJ) to a final concentration of 1%, and tubes were rocked at RT for 7 min. Cross-linking was stopped by adding 10× glycine (1.5 ml), and the tubes were again rocked at RT for 7 min. The ChIP-IT Express Kit (53008, Active Motif, Carlsbad, CA) was employed according to manufacturer’s instructions. Briefly, (1) cells were pelleted by centrifugation for 10 min at 2500 rpm at 4 °C; (2) cells were washed twice with ice-cold PBS; (3) cells were lysed with 600 µl ice-cold lysis buffer (supplemented with 6 µl protease inhibitor cocktail + 6 μl PMSF); (4) tubes were briefly vortexed and rotated at 4 °C for 30 min; (5) lysates were spun at 5000 rpm for 10 min at 4 °C to pellet nuclei; (6) nuclei pellet was resuspended in 400 µl shearing buffer (supplemented with 4 μl protease inhibitor cocktail + 4 μl PMSF); (7) chromatin was sheared with a ultrasonic pressure (model CP750, Cole-Parmer Instruments, Illinois, USA) using the following settings: 60% Amplitude; 40 s per cycle; total cycle for 10 in ice; (8) sheared chromatin samples were spun at 15,000 rpm at 4 °C for 10 min; and (9) supernatant was collected and aliquoted as chromatin samples. For IP process, anti-NF-κB2 (Cell signaling technology), anti-Pol II and control Igs (ChIP-IT control kit, 53027, Active Motif, Carlsbad, CA) were employed and listed in Supplementary Table 1. Each IP experiment used 3 μg Abs and was performed using the reagents and protocol provided by the ChIP-IT Express Kit according to manufacturer’s instructions. For quantitative PCR (qPCR), samples were purified with a ChIP DNA clean and concentrator kit (D5205, Zymo Research). Templates and primers were added into the LightCycler® 480 SYBR Green I Master (04707516001, Roche, Basel, Switzerland) in a total volume of 10 μl per reaction. The reaction was run on Light Cycler 480II (Roche, Basel, Switzerland). The PCR conditions and primers are listed in Supplementary Table 2. ChIP-qPCR data were normalized based on the notion that IP of a specific Ab contains both specific signal and background, whereas IP of a negative control-Ig contains background only. The background/input was substracted from signal/input, and the remaining value corresponded to net pull-down of a specific DNA/chromatin region by a given specific Ab normalized to total chromatin and nonspecific (negative control) IP.

### Real-time PCR

Purified B cells of LMC, B-TRAF2/3-DKO, B-TRAF3-KO and B-TRAF3/NF-κB2-DKO were treated with Ara-C for 16 h. Total RNA was purified with Trizol reagent (15596026, Invitrogen). For each cDNA synthesis reaction, total template RNA (500 ng) was used according to manufacturer’s instructions using Revert Aid First Strand cDNA Synthesis Kit (K1622, Thermo Scientific) followed by qPCR. Briefly, the reaction mixture contained 4 μl of diluted cDNA (1:20) sample, 0.2 μM Primers and 5 μl of SYBR green mix. PCRs were performed in Light Cycler 480II (Roche, Basel, Switzerland). The primers and PCR reaction condition were listed in Supplementary Table 2. For relative quantification, β-actin was employed to normalize experimental variation and avoid any possible experimental errors. Comparative C_t_ method (2^−ΔΔCt^) was employed to calculate fold change of expression. The C_t_ values of the respective genes were normalized with C_t_ values of β-actin in their respective samples. A negative control was also included with each set of reactions, which contains all PCR reagents other than cDNA.

### IgH VDJ sequencing and Spectral Karyotyping (SKY)

Five samples (383 C, 521 C, 313 C, 391 C, 264 C) were sequenced by ImmunoSEQ platform for IgH CDR3 region and VDJ usage (Adaptive Technologies). ImmunoSEQ Analyzer was used to retrieve, process, and track the *Igh* sequencing data. The data retrieved from immunoSEQ analyzer contained two columns that are particularly relevant to BCR repertoire analysis, “Productive Frequency” representing the frequency of each productive BCR DNA sequence obtained per sample and “Productive Rearrangements” (Seq#) representing the number of unique productive BCR DNA sequences in each sample. For pareto plot analysis, we combined the productive rearrangements that encode the same CDR3 amino acid (a.a.) and contain the same V, D, J gene usage into the same BCR clonotypes using R (version 4.1.0).

Diseased B cells (383 C, 521 C, 313 C, 391 C, 264 C) or primary B cells (LMC and B-TRAF2/3-DKO) were analyzed by SKY. Briefly: (1) diseased B cells (0.5 × 10^6^/ml, 6 ml/well in 6-well plate) were cultured and treated with 100 ng/ml colcemid (15212-012, Gibco KaryoMAX) for 4 h; (2) cells were harvested, washed in PBS, resuspended in 75 mM KCl and incubated at RT for 10–15 min; (3) fixation buffer (3:1 ratio of Methanol: Acetic acid) (¼ volume) was added into cells and mixed gently; (4) fixed cells were centrifuged at 800 rpm for 5 min; (5) supernatant was discarded and cells were resuspended in fixation buffer and held for 48 h at −20 °C. After 48 h, metaphase slides were prepared. Briefly: (1) cells were spun at 1500 g for 5 min and resuspended in fresh fixation buffer; (2) cell suspension (10–20 μl) was dropped to the center of a slide (Superfrost® Plus White, Cat 48311-703, VWR). When the front of solution spread, slide was inverted over the water bath (83 °C) for 5–10 s; (3) slides were dried on a metal plate kept in water bath for 30 s and additional 2 mins on a heating plate (80 °C); (4) the quality of metaphase spreads was evaluated under light microscope (Olympus CKX41); and (5) slides were aged at RT for 2 days and submitted for SKY (Applied Spectral Imaging (ASI)). To detect the chromosomal aberrations in primary B cells, purified B cells (0.5 × 10^6^/ml, 6 ml/well in 6-well plate) from LMC and B-TRAF2/3-DKO mice were stimulated with LPS (5 μg/ml) and IL-4 (10 ng/ml) for 4 days. After 4 days, cells were treated with colcemid, metaphase spreads were prepared and submitted for SKY as described above. The SKY hybridization was performed according to the regular SKY protocol (ASI). Briefly, the SKY probe containing all 21 labeled mouse chromosome libraries was hybridized simultaneously to the slides containing the fixed metaphases. After washing, the slides were stained with 4'6-diamidino-2 phenylindole (DAPI) in anti-fade medium. Selected metaphases were captured and analyzed using special software for acquisition and analysis of chromosomes (Hi-SKY, ASI). The DAPI images were captured separately and inverted to give a G-banding like pattern. The chromosomes were then sorted automatically into a karyotype table and analyzed for the existence of any structural or numerical aberrations. From each mouse sample 12–14 metaphases were randomly chosen for full karyotype and analysis.

### Statistical analysis

All multiple group data comparison employed one-way or two-way ANOVA analysis. Two group data comparison employed Unpaired Student’s *T* test, Multiple *T* test or other tests indicated in the figure legend. Data were shown as mean±sem. GraphPad Prism 9.1.4 software (GraphPad Software, Inc.) was used to analyze data, *p* < 0.0001 as very very very significant (****), *p* < 0.001 as very very significant (***), *p* < 0.01 as very significant (**), and *p* < 0.05 as significant (*).

## Results

### TRAF2/3 double deficient B cells were more resistant to DNA damage-induced apoptosis

**Fig. 1:**
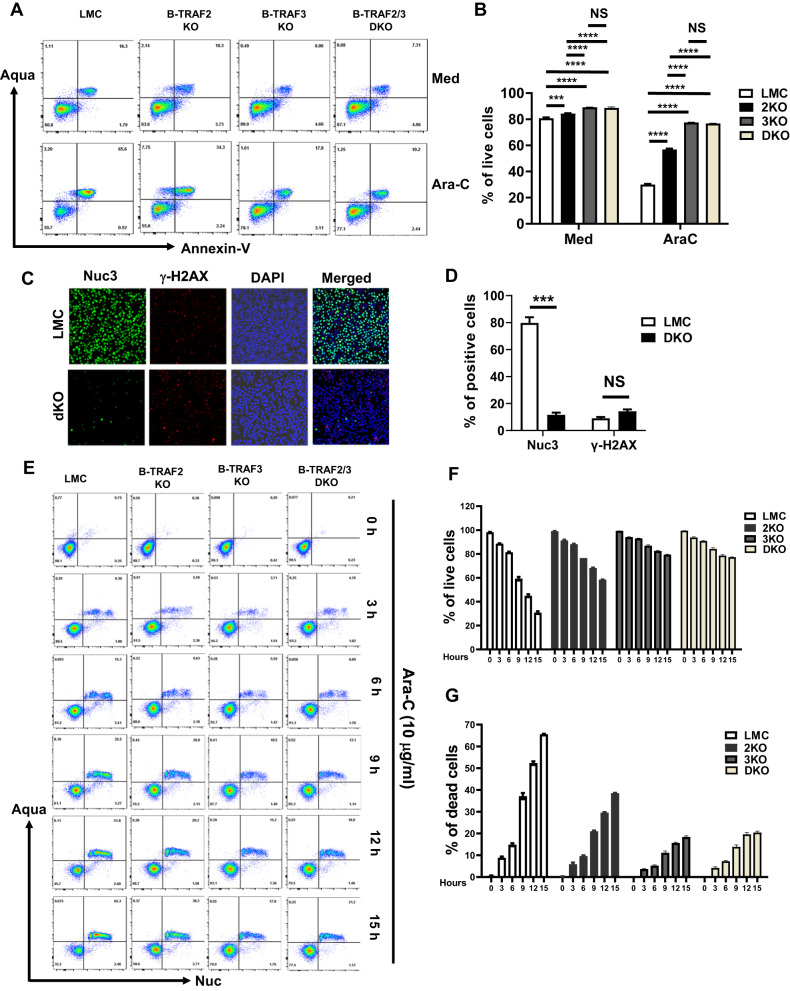
TRAF2/3 double deficiency protects B cells from DNA damage-induced apoptosis. **A** Representative flow cytometry data of B cells cultured for 16 h in the absence (Med) or presence of Ara-C. Dead cells: Aqua^+^ or Aqua^+^Annexin-V^+^, apoptotic cells: Annexin V^+^; alive cells: Aqua^−^Annexin-V^−^. LMC: littermate control; Med: medium; Ara-C: Cytarabine. All abbreviations are applied to other panels. **B** Quantification of the percentage of alive cells from triplicates of one representative experiment described in panel A. 2KO: B-TRAF2-KO, 3KO: B-TRAF3-KO, DKO: B-TRAF2/3-DKO. **C** Representative fluorescent microscopy data of LMC or DKO B cells treated by Ara-C in the presence of caspase3 substate (Nuc3) for 16 h. Cells were stained by anti-γ-H2AX Ab and DAPI. Nuc3: green signal for active caspase3, γ-H2AX: red signal for DSBs; DAPI: blue signal for nuclei. 40× magnification. **D** Quantification of the percentage of positive cells for Nuc3 or γ-H2AX. **E** Representative flow cytometry data showing the kinetics of real-time apoptosis. LMC, B-TRAF2-KO, B-TRAF3-KO or B-TRAF2/3-DKO B cells were treated by Ara-C and caspase3 substate (Nuc3) for indicated time points and stained by Aqua. **F**, **G** Quantification of the percentage of alive (**F**) or dead (**G**) cells from triplicates of one representative experiment described in panel E. Statistical analysis was performed using unpaired student’s T test for panel D, or two-way ANOVA for panel B, F and G. Significant difference was detected between LMC and B-TRAF2-KO, B-TRAF3-KO or B-TRAF2/3-DKO B cells for all timepoints of treatment (3 h to 15 h), ***p < 0.001, no significant difference detected for 0 h (no treatment), and no significant difference detected between B-TRAF3-KO and B-TRAF2/3-DKO B cells. All experiments were repeated independently 3 or 5 times.

It has been previously reported that B-TRAF2-KO or B-TRAF3-KO B cells have longer lifespan than their corresponding littermate control (LMC) [24]; however, it remains unknown whether B-TRAF3-KO (CD19Cre-TRAF3^f/f^), B-TRAF2-KO (CD19Cre-TRAF2^f/f^), or B-TRAF2/3-DKO (CD19Cre-TRAF2^f/f^/TRAF3^f/f^) B cells are more capable of tolerating DNA damage. To test whether single deficiency of TRAF3 or TRAF2 or double deficiency protects B cells against DNA damage-induced apoptosis, we isolated splenic B cells from LMC (TRAF2^f/f^/TRAF3^f/f^), B-TRAF3-KO, B-TRAF2-KO or B-TRAF2/3-DKO mice and cultured B cells in the absence or presence of cytarabine (Ara-C), a chemotherapeutic drug that causes cell death by inducing DNA double-stranded breaks (DSBs). We performed flow cytometry of Aqua vs. Annexin-V staining to assess cell viability (alive cells: Aqua^−^Annexin-V^−^; apoptotic cells: Annexin-V^+^; dead cells: Aqua^+^ or Aqua^+^Annexin-V^+^). In the absence of Ara-C, LMC B cells survived well with ∼80% alive cells, whereas Ara-C treatment reduced the percentage of alive cells to ∼30% in LMC B cells (Fig. 1A, B, 80.8% in Med vs. 30.7% in Ara-C). In the presence of Ara-C, B-TRAF2-KO, B-TRAF3-KO and B-TRAF2/3-DKO B cells survived much better than LMC (Fig. 1A, B). Moreover, B-TRAF3-KO and B-TRAF2/3-DKO B cells survived better than B-TRAF2-KO ones, while there was no statistical difference between B-TRAF3-KO and B-TRAF2/3-DKO B cells (Fig. 1A, B). These data show that single deficiency of TRAF3 or TRAF2 or double deficiency protects B cells against DNA damage-induced apoptosis.

To further corroborate our data and visualize real-time apoptosis in the presence of DNA damage, we treated LMC and B-TRAF2/3-DKO B cells with Ara-C in the presence of Nuc3, a newly developed substrate of active caspase 3 that can detect caspase-mediated apoptotic cells (see details in Method). Using fluorescent microscopy, we showed that the percentage of Nuc3^+^ cells was much higher in LMC than B-TRAF2/3-DKO B cells in the presence of Ara-C (Fig. 1C, D), demonstrating a higher level of real-time apoptosis in LMC than B-TRAF2/3-DKO B cells. We also measured the level of γ-H2AX, an indicator of DSB formation, upon Ara-C treatment to test whether the level of DSB generation differed. We found that the level of γ-H2AX was not statistically different between LMC and B-TRAF2/3-DKO B cells (Fig. 1C, D), suggesting that LMC B cells did not have more DSBs generated. While the level of DSBs did not differ significantly, B-TRAF2/3-DKO B cells survived much better than LMC upon Ara-C treatment, further supporting the notion that TRAF2/3 double deficiency attenuates DNA damage-induced apoptosis.

To delineate the kinetics of real-time apoptosis, we examined the percentage of alive vs. dead cells in a time dependent manner by performing Aqua vs. Nuc3 staining using LMC, B-TRAF2-KO, B-TRAF3-KO and B-TRAF2/3-DKO B cells at various time points upon Ara-C treatment. At 0 h, LMC, B-TRAF2-KO, B-TRAF3-KO and B-TRAF2/3-DKO B cells had a similar percentage of alive cells (Aqua^−^Nuc^−^) (Fig. 1E). Upon Ara-C treatment, LMC B cells exhibited time-dependent real-time apoptosis (Fig. 1E–G). In contrast, by 15 h after Ara-C treatment, the majority of B-TRAF3-KO and B-TRAF2/3-DKO B cells (∼80%) were still alive, while the percentage of apoptotic cells did increase, the extent of the increase was much lower than LMC B cells (Fig. 1E–G). The phenotypes of B-TRAF2-KO B cells were between LMC and B-TRAF3-KO or B-TRAF2/3-DKO ones (Fig. 1E–G). These results demonstrate that real-time caspase-3 activation in LMC B cells causes cell death whereas caspase-3 is less activated in B-TRAF2-KO, B-TRAF3-KO, or B-TRAF2/3-DKO B cells at all time points tested. We suggest that either single deficiency or double deficiency of TRAF2/3 can continuously protect B cells in the presence of DNA damage by attenuating apoptosis; furthermore, TRAF3 single deficiency can protect B cells as well as TRAF2/3 double deficiency.

### TRAF2/3 double deficiency reduces caspase activation and promotes transcription of caspase inhibitors

**Fig. 2:**
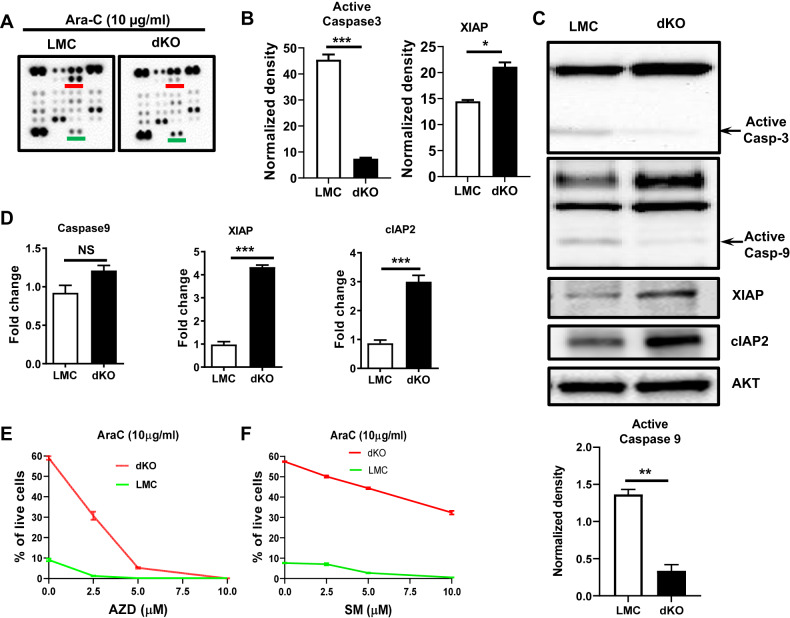
TRAF2/3 double deficiency reduces caspase activation and promotes transcription of IAPs that protect B-TRAF2/3-DKO B cells from DNA damage-induced apoptosis. **A** Representative data of proteome profiler mouse apoptosis array of LMC or dKO (B-TRAF2/3-DKO) B cells cultured for 16 h in the presence of Ara-C (10 μg/ml). Red line: active caspase3; green line: XIAP. All abbreviations are applied to other panels. **B** Quantification of dot density of active caspase 3 and XIAP presented in panel A. **C** Representative data of western blotting of LMC or DKO B cells treated by Ara-C for 16 h. Casp-3: caspase 3, Casp-9: caspase 9. AKT as protein loading control. Quantification of the band intensity for active Caspase 9 in LMC or DKO B cells (*n* = 2). **D** Quantification of real-time PCR data from triplicates of one representative experiment using LMC or dKO B cells treated by Ara-C for 16 h. Statistical analysis was performed using unpaired student’s *t* test for panel B and D. **E**, **F** Quantification of the percentage of alive cells upon IAP antagonist treatment. LMC or dKO B cells were pretreated with indicated concentration of a pan IAP inhibitor, AZD5582 (AZD) (**E**) or a XIAP selective inhibitor, SM-164 (SM) (**F**), then cultured in the presence of Ara-C for 16 h. For panel E and F, cells were stained by Aqua and Annexin-V for flow cytometry analysis. Quantification of alive cells are shown from duplicates of one experiment of flow data. Statistical analysis was performed using two-way ANOVA for panel E and F. Significant difference was observed between LMC and dKO for all concentration (0 to 5 μM) except 10 μM for panel E, and for all concentration (0 to 10 μM) for panel F. NS no significance (*p* > 0.05); *p < 0.05, ***p < 0.001. All experiments were independently repeated 2 to 5 times.

To elucidate the mechanism by which TRAF2/3 regulate apoptosis pathway upon DNA damage, we employed proteome profiler mouse apoptosis array to detect apoptosis-related protein expression or activation in Ara-C treated LMC or B-TRAF2/3-DKO B cells. Our data showed that activated caspase 3 was significantly higher in LMC than B-TRAF2/3-DKO B cells (Fig. 2A, B). In contrast, B-TRAF2/3 DKO B cells express a significantly higher level of XIAP (X-linked inhibitor of apoptosis) than LMC ones (Fig. 2A, B). XIAP can inhibit caspase 3 activation by blocking caspase 9 activity. We validated our array results using western blotting that confirmed reduced caspase 3 activation and increased XIAP expression in B-TRAF2/3-DKO B cells compared to LMC ones (Fig. 2C). The apoptosis array kit did not include caspase 9 which initiates intrinsic apoptosis pathway. Using western blotting, we found that activation of caspase 9 was reduced in B-TRAF2/3-DKO B cells compared with LMC (Fig. 2C). Caspase 9 activation can be inhibited by cIAP2 (a.k.a. BIRC3). Consistently, we found the level of cIAP2 protein expression was higher in B-TRAF2/3-DKO B cells than LMC (Fig. 2C). Next, we used Real-time PCR to examine the transcription of caspase 9, XIAP and cIAP2. LMC and B-TRAF2/3-DKO B cells expressed a comparable level of caspase 9 transcripts, whereas B-TRAF2/3-DKO B cells expressed a significantly higher level of XIAP and cIAP2 transcripts (Fig. 2D), consistent with increased XIAP and cIAP2 protein expression (Fig. 2C).

Next, we addressed whether XIAP and cIAP2 both play a role in protecting B-TRAF2/3-DKO B cells from DNA damage-induced apoptosis. To do so, we pretreated B-TRAF2/3-DKO or LMC B cells with different concentrations of SM-164 (a selective inhibitor for XIAP) or AZD5582 (an inhibitor for both XIAP and cIAP2), then, cultured them in the presence of Ara-C (Fig. 2E, F). Without IAP inhibitor treatment, B-TRAF2/3-DKO B cells survived much better than LMC ones in the presence of Ara-C. The enhanced survival of B-TRAF2/3-DKO B cells depended on both XIAP and cIAP2 since AZD5582 strongly promoted Ara-C-induced cell death in B-TRAF2/3-DKO B cells in a dose-dependent manner (Fig. 2E, Supplementary Fig. 1). SM-164 also promoted Ara-C-induced cell death in B-TRAF2/3-DKO B cells, albeit to a lesser extent (Fig. 2F, Supplementary Fig. 1). Hence, we conclude that both XIAP and cIAP2 are critical for protecting B-TRAF2/3-DKO B cells from DNA damage-induced apoptosis.

### NF-κB2 controls the transcription of XIAP and cIAP2 and is required for enhanced B cell survival upon DNA damage-induced apoptosis

**Fig. 3:**
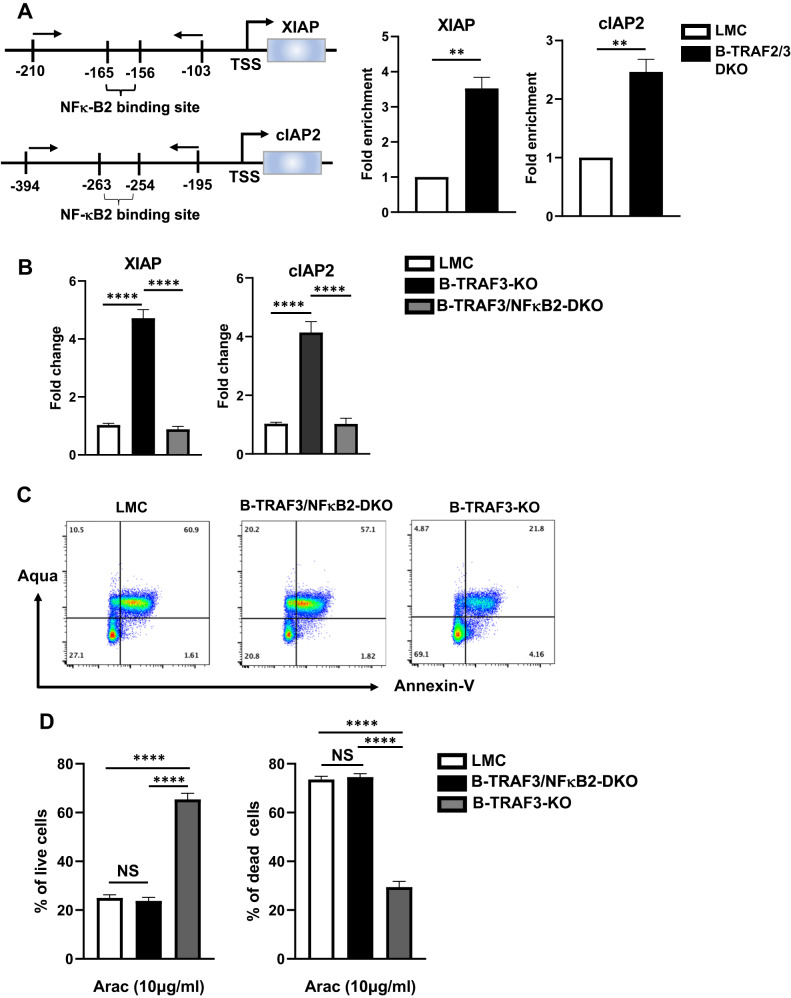
NF-κB2 controls the transcription of XIAP and cIAP2 and is required for enhanced B cell survival upon DNA damage-induced apoptosis. **A** Left: Schematics of NF-κB2 binding sites in the promoter region of XIAP and cIAP2 genetic loci. Arrows indicate the positions of the primers used for ChIP-qPCR. Right: ChIP-qPCR data of the enhanced fold enrichment of NF-κB2 binding at XIAP and cIAP2 promoters. Three independent experiments were performed and representative data from one experiment is shown. Statistical analysis was performed by unpaired student *t* test. **B** Transcription level of XIAP or cIAP2 in the B cells of LMC, B-TRAF3-KO and B-TRAF3/NF-κB2-DKO mice. B cells were purified and cultured in the presence of Ara-C for 16 h. Representative of triplicated quantitative PCR data is shown. Data are statistically analyzed by one-way ANOVA. **C** Representative flow cytometry data of B cells of LMC, B-TRAF3/NF-κB2-DKO or B-TRAF3-KO mice. B cells were cultured for 16 h in the presence of Ara-C and stained with Aqua vs. Annexin-V. **D** Quantification of the percentage of alive (Left) and dead (Right) cells of triplicated flow data in panel C. Alive cells: Aqua^−^Annexin-V^−^; apoptotic cells: Annexin-V^+^; dead cells: Aqua^+^ or Aqua^+^Annexin-V^+^. Data were statistically analyzed by one-way ANOVA. All experiments were independently repeated 3 times. NS not significant (*p* > 0.05); *p < 0.05; **p < 0.01; ****p < 0.001.

XIAP and cIAP2 transcription was increased in B-TRAF2/3-DKO B cells that exhibited a higher level of NF-κB2 activation. Thus, we tested whether NF-κB2 directly regulates XIAP and cIAP2 transcription using Chromatin Immunoprecipitation (ChIP) assay. We employed LMC or B-TRAF2/3-DKO B cells cultured in the presence of Ara-C to perform ChIP assay using anti-NF-κB2 antibody. NF-κB2 binding sites were shown in the promoter region of XIAP and cIAP2 genetic loci (Fig. 3A, left). Our data showed that the binding of NF-κB2 to the promoter region of XIAP and cIAP2 was significantly increased in B-TRAF2/3-DKO B cells compared to LMC (Fig. 3A, right). These results suggest that NF-κB2 directly upregulates the transcription of XIAP and cIAP2 in B-TRAF2/3-DKO B cells.

To definitively test the role of NF-κB2 in upregulating XIAP and cIAP2, we generated B-TRAF3/NF-κB2-DKO (CD19Cre-TRAF3^f/f^/NF-κB2^f/f^) mice, and cultured B cells isolated from LMC, B-TRAF3-KO or B-TRAF3/NF-κB2-DKO mice in the presence of Ara-C. Consistent with phenotypes of B-TRAF2/3-DKO B cells (Fig. 2D), B-TRAF3-KO B cells expressed a significantly higher level of XIAP and cIAP2 transcripts than LMC (Fig. 3B). The increased XIAP and cIAP2 transcription was completely dependents on NF-κB2 since B-TRAF3/NF-κB2-DKO B cells failed to upregulate XIAP and cIAP2 transcripts (Fig. 3B). Next, we tested whether enhanced B cell survival upon Ara-C treatment also required NF-κB2. We performed flow cytometry of Aqua vs. Annexin-V staining to assess the viability of LMC, B-TRAF3-KO or B-TRAF3/NFκB2-DKO B cells in the presence of Ara-C. Consistent with phenotypes of B-TRAF2/3-DKO B cells (Fig. 1A, B), B-TRAF3-KO B cells survived significantly better than LMC ones (Fig. 3C, D). The enhanced B cell survival was also completely dependent on NF-κB2 since there was no significant difference between LMC and B-TRAF3/NF-κB2-DKO B cells in their percentage of alive or dead cells upon Ara-C treatment (Fig. 3C, D). We conclude that NF-κB2 is required for upregulated XIAP and cIAP2 transcription as well as enhanced B cell survival upon DNA damage-induced apoptosis in the absence of TRAF3.

### B cell-specific double deficiency of TRAF2/3 shortens lifespan but does not accelerate or enhance B-cell lymphomagenesis

**Fig. 4:**
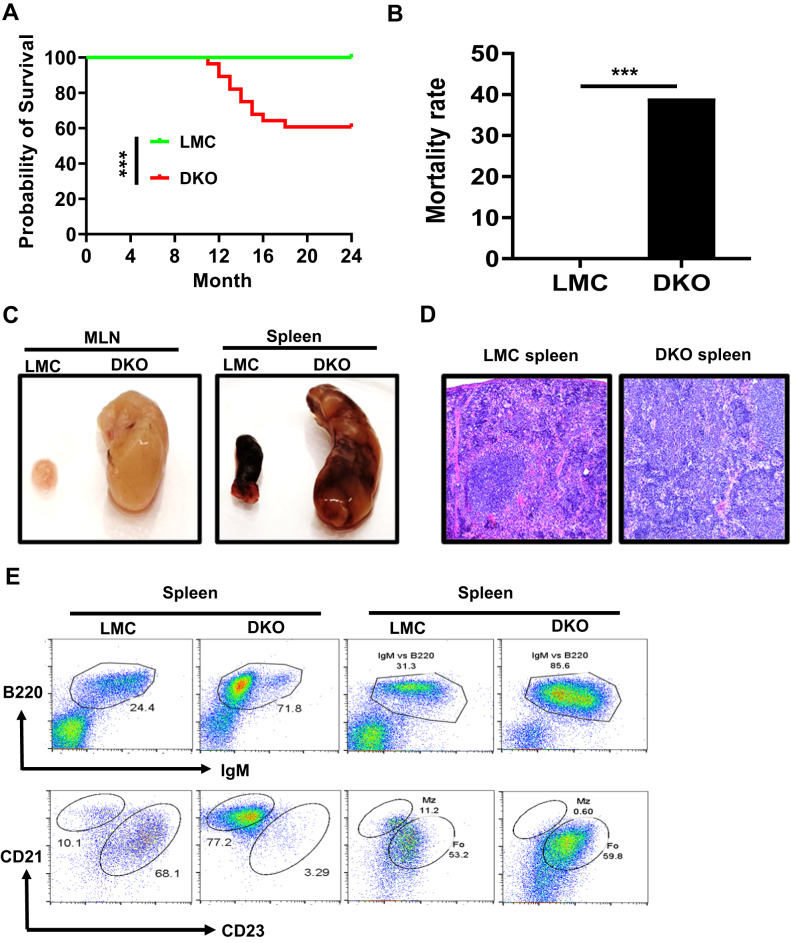
B cell-specific TRAF2/3 double deficiency causes lymph organ abnormality and reduces mouse survival. **A** Kaplan-Meier survival curve: percent survival of LMC (*n* = 28) and B-TRAF2/3-DKO mice (*n* = 28) versus age in months is shown. Statistical significance was compared between two groups using log rank (Mantel-Cox test). ***p < 0.001. **B** Total mortality rate of mice described in panel (**A**). All mice were observed for 24 months. Morbid mice were euthanized according to IACUC guidelines. Statistical significance was compared between two groups by Chi-squared test, ***p < 0.001. **C** Representative image of splenomegaly and lymphadenopathy. Left: Comparison of mesenteric lymph nodes (MLN) of LMC and dKO (B-TRAF2/3-DKO) mice. Right: Comparison of LMC and dKO mouse spleens. **D** Representative image of hematoxylin and eosin (HE) analysis of LMC and dKO mouse spleens. 20× magnification. Data from panel (**C**) and (**D**) were independently repeated more than 3 times. **E** Representative flow cytometry data of LMC spleen (*n* > 10) and diseased dKO (*n* > 10) mice with extremely enlarged spleens. Upper: B220 vs. IgM, Lower: marginal zone (MZ) and follicular (FO) B cell profiling.

To test whether TRAF2/3 double deficiency accelerates or enhances B-cell lymphomagenesis, we established a cohort of B-TRAF2/3-DKO and LMC mice. We found that B-TRAF2/3-DKO mice exhibited a significantly shorter lifespan compared to LMC mice (Fig. 4A). About 40% of aged B-TRAF2/3-DKO mice (>11 months of age) developed lymphoid organ abnormality including splenomegaly and lymphadenopathy manifested as extremely enlarged spleen and lymph nodes or over-burden ascites (Fig. 4B, C, Supplementary Table 3). Diseased mice became morbid, which eventually required euthanasia according to IACUC guidelines. Hematoxylin and eosin (H&E) staining showed that splenic red and white pulp structure was almost completely disrupted by lymphocyte expansion in B-TRAF2/3-DKO samples (Fig. 4D). Consistently, diseased B-TRAF2/3-DKO mice exhibited abnormal B cell expansion in spleen (Supplementary Fig. 2).

Previous studies showed that both follicular (FO) and marginal zone (MZ) B cells were increased in B-TRAF3-KO mice, while MZ B cells were preferentially expanded [5, 22]. Hence, we performed flow cytometry to analyze B cell populations in the extremely enlarged spleens of B-TRAF2/3-DKO mice. All of the examined B-TRAF2/3-DKO mice contained expanded B cell populations compared to LMC (Fig. 4E, top, B220^+^IgM^+^, Supplementary Fig. 2A). Some of B-TRAF2/3-DKO mice predominantly expanded MZ B cells, whereas others only expanded FO B cells and lacked MZ B cells (Fig. 4E, bottom, CD21 vs. CD23, Supplementary Fig. 2B). In most of B-TRAF2/3-DKO mice, splenic FO and MZ B cell populations varied between the two extreme scenarios, implying that B cell expansion in B-TRAF2/3-DKO mice occurred in a stochastic manner.

We next examined whether the highly expanded B cells have lymphoma characteristics. We first tested whether they could be continuously cultured in vitro as we have done previously to establish G1XP lymphomas [31]. Within a cohort of 28 aged B-TRAF2/3-DKO mice, we tested B cell samples from 11 mice with an extremely enlarged spleen or ascites (Supplementary Table 3). Only 1 splenic B cell sample (264 C) could be continuously cultured in vitro, indicating that other 10 samples did not exhibit a full-blown malignant phenotype. Next, we tested their tumorigenesis potential in vivo by transplanting expanded B-TRAF2/3-DKO B cells into immunocompromised mice. Only 264 C B cells could grow tumors in immunocompromised mice two months after transplantation (Supplementary Table 3), further suggesting those highly expanded B-TRAF2/3-DKO B cells are not tumorigenic. We conclude that B-TRAF2/3-DKO mice succumbed to lymphoid organ abnormality frequently. Our results indicate that TRAF2/3 double deficiency does not accelerate or enhance B-cell lymphomagenesis compared to TRAF3 single deficiency [23].

### TRAF2/TRAF3 double deficiency leads to oligoclonal and clonal expansion of B cells

**Fig. 5:**
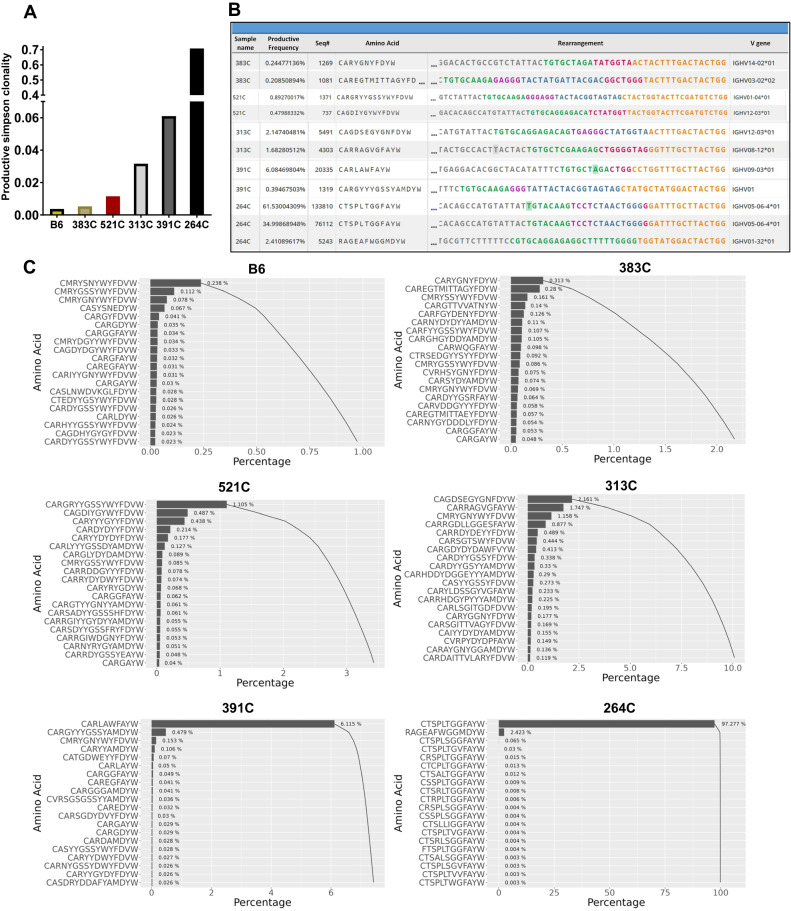
Oligoclonal and clonal expansion in TRAF2/3 double deficient B cells. **A** Productive Simpson clonality showing the extent of clonal expansion in sequenced B cell samples (*n* = 5) with WT B6 spleen as control. **B** The top 2 expanded BCR (*Igh*) clonotypes are shown for each sample (*n* = 5), including productive frequency, the number of total productive templates (Seq#), CDR3 amino acid (a.a.), CDR3 rearrangement and V gene usage. Green color: start of CDR3 in V-gene; violet color: N1 junction; blue color: D-gene; pink color: N2 junction; yellow color: J-gene. **C** Pareto plots of sequencing data to show the cumulative percentage of top 20 BCR (*Igh*) clonotypes in each sample including WT B6 as a control. Each clonotype shown contains the same CDR3 a.a. and VDJ gene usage.

To test whether mutant B cells undergo any level of clonal expansion, we performed *Igh* CDR3 DNA sequencing using Adaptive Biotechnologies’ immunoSEQ platform. We sequenced 5 genomic DNA samples isolated from purified B cells of 5 individual aged diseased B-TRAF2/3-DKO mice (383 C, 521 C, 313 C, 391 C, 264 C). Productive Simpson clonality was calculated to determine the extent of clonal expansion in sequenced samples (Fig. 5A). Values of Simpson clonality range from 0 (polyclonal) to 1 (monoclonal). Four out of five sequenced samples (383 C, 521 C, 313 C, 391 C) showed values ranging from 0.0053 to 0.061, indicating oligoclonal expansion, whereas 264 C showed a value of 0.7083, suggesting monoclonal expansion. The top 2 expanded BCR clonotypes were shown for all 5 samples including productive frequency, the number of total productive templates (Seq#), CDR3 amino acid (a.a.), CDR3 rearrangement and V gene usage (Fig. 5B).

Pareto plots showed the cumulative percentage of top 20 clonotypes in each sample including WT B6 as a negative control (Fig. 5C). B6 sample has a minimal level of clonal expansion evidenced by only two clonotypes with frequency of >0.1% (Fig. 5C, Supplementary Fig. 3). Four of five samples, 383 C, 521 C, 313 C, 391 C, contained predominantly expanded clonotypes with a frequency of 0.313%, 1.105%, 2.161% and 6.115%, respectively. We found that 264 C showed the clonal expansion of a predominant clonotype CTSPLTGGFAYW with a frequency of 97.277%. This is the cumulative frequency of all clonotypes that have the same CDR3 a.a. and VDJ gene usage but contain nucleotide substitutions in their V gene segment, such as the two clonotypes shown in Fig. 5B for 264 C (61.530% and 34.999%). Our data demonstrate that 264 C sample contained clonally expanded B cell populations, namely, lymphoma cells.

The clonal frequency for each top-expanded clonotype (defined as >0.1%) was calculated and presented as the percentage of the entire *Igh* repertoire sequenced (Supplementary Fig. 3). Consistently, samples of 383 C, 521 C, 313 C, and 391 C showed oligoclonal expansion with multiple clonotypes >0.1%. In contrast, 264 C showed the major population of expanded clonotype with a frequency of 97.28%, while the percentage of clonotypes (<0.1%) in 264 C was found to be only 0.3%. Taken together, our data showed that TRAF2/3 double deficiency resulted in oligoclonal or clonal expansion of B cells.

### Diseased TRAF2/3 double deficient B cells harbor chromosomal alterations

**Fig. 6:**
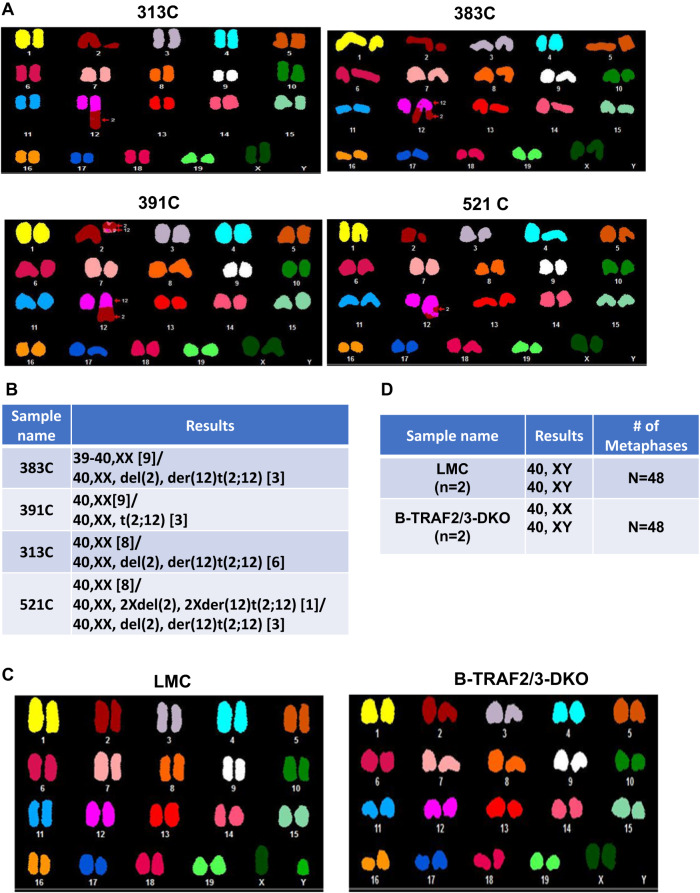
SKY analysis in diseased and non-diseased TRAF2/3 double deficient B cells. **A** Representative SKY data showing the chromosomal alterations in diseased B-TRAF2/3-DKO mice (*n* = 4). **B** Summary of the number and type of translocations observed in analyzed metaphases (*n* = 12–14 metaphases per sample). **C** Representative SKY data showing no detectable chromosomal alterations in primary B cells from LMC (*n* = 2) or non-diseased B-TRAF2/3-DKO mice (*n* = 2). **D** Summary of the number of metaphases analyzed and normal karyotyping in all the samples (*n* = 24 metaphases per mice).

Chromosomal alterations implicate genomic instability and chromosomal translocation is a hallmark of human B cell lymphomas [31, 32]. To test whether TRAF2/3 double deficiency enhances genomic instability in B cells, we isolated B cells from 5 diseased B-TRAF2/3-DKO mice with splenomegaly and lymphadenopathy or ascites and prepared metaphases from 5 samples. Metaphase slides were subjected to Spectral Karyotyping (SKY) with Applied Spectral Imaging (ASI) to determine chromosomal alterations. We observed unbalanced translocations in 313 C, 383 C, 521 C and balanced reciprocal translocations in 391 C, all of which involved chromosomes 2 and 12 (Fig. 6A). Of note, *Igh* locus is located at the telomeric region of chromosome 12. The percentage of metaphases containing translocations ranged from 25% to 43% in the samples of 383 C (25%, *n* = 3/12 metaphases), 391 C (25%, *n* = 3/12 metaphases), 313 C (43%, *n* = 6/14 metaphases), and 521 C (33%, *n* = 4/12 metaphase), respectively (Fig. 6B). Intriguingly, 264 C B cells exhibited drastic clonal expansion but had no detectable chromosomal alterations (Supplementary Fig. 4). We suggest that such chromosomal alterations detected can indicate genomic instability but are not required for lymphoma development in B-TRAF2/3-DKO B cells. We cannot exclude the possibility that other genetic mutations caused B cell malignancy in 264 C.

Next, we tested whether non-diseased primary B cells of young B-TRAF2/3-DKO mice (8 weeks) harbored chromosomal alterations. We stimulated the purified B cells of LMC (TRAF2^f/f^TRAF3^f/f^) (*n* = 2) or B-TRAF2/3-DKO (CD19Cre-TRAF2^f/f^TRAF3^f/f^) (*n* = 2) with LPS and IL-4 for 4 days. Metaphase spreads were prepared from stimulated B cell samples and subjected to SKY analysis. In total, 48 metaphases were analyzed from either LMC or B-TRAF2/3-DKO samples and no chromosomal alterations were observed in any sample (Fig. 6C, D). Taken together, we conclude that TRAF2/3 deficiency per se does not lead to genomic instability in B cells at an appreciable level; instead, it may permit a fraction of B cells which harbor DNA damage to survive and accumulate genetic alternations gradually.

### Establishing a TRAF2/3 double mutant lymphoma model and increased sensitivity to IAP antagonist in TRAF3-mutant lymphomas

**Fig. 7:**
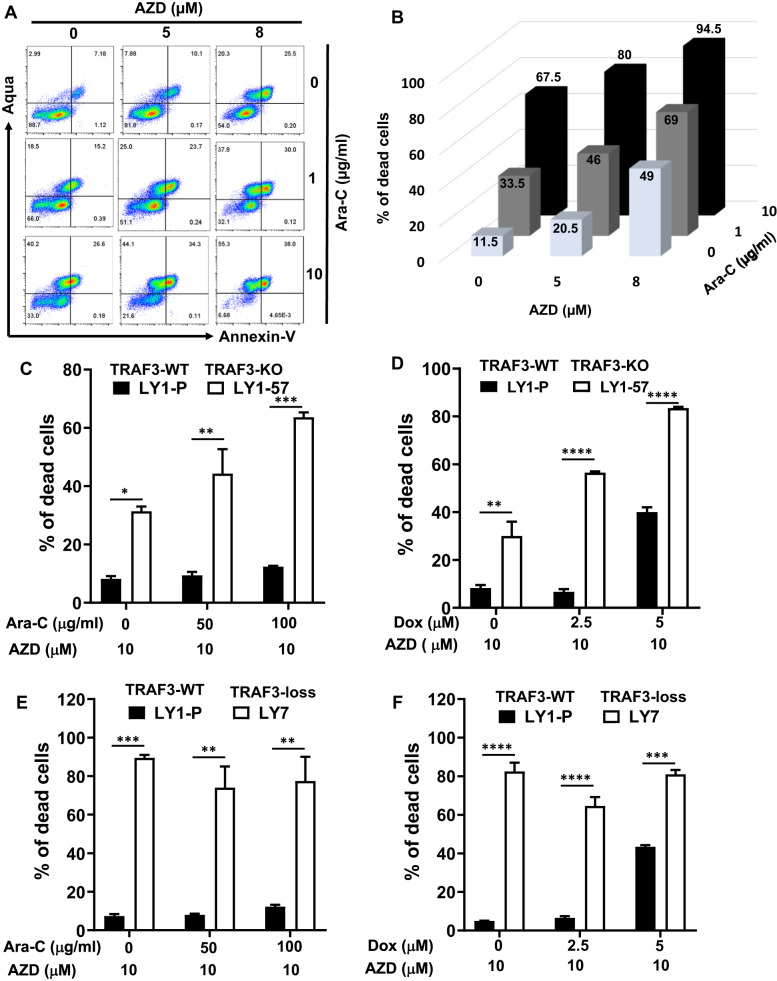
Targeting IAP activity enhances sensitivity of TRAF2/3 double or TRAF3 single deficient lymphoma cells to genotoxin-induced cell death. **A** Representative flow cytometry data of our newly established TRAF2/3 double deficient 264 C mouse lymphoma cells. Cells were pretreated with or without a pan IAP inhibitor AZD5582 (AZD), cultured for 16 h in the absence or presence of Ara-C with indicated concentrations, and stained with Aqua and Annexin-V. **B** Quantification of the percentage of dead cells from duplicated flow data in panel A. Numbers in each column indicate the mean percentage for each condition. **C**–**F** Quantification of the percentage of dead cells from duplicated flow cytometry data of human B cell lymphomas. Human B cell lymphomas were cultured for 16 h in the presence of indicated concentrations of Ara-C or doxorubicin (Dox) with AZD. LY1-P: LY1 parental cells (TRAF3-WT); LY1-57: LY1 clone whose TRAF3 is deleted by gRNA/CRISPR/Cas9 (TRAF3-KO); LY7: LY7 parental cells expressing no detectable TRAF3 protein (TRAF3-loss). Flow cytometry analysis was performed as described in methods. Data are statistically analyzed by two-way ANOVA, *p < 0.05; **p < 0.01; ***p < 0.001, ****p < 0.0001. All experiments were independently repeated 3 times.

We next tested whether 264 C lymphoma cells were sensitive to chemotherapeutic drugs or IAP antagonists such as AZD5582. We treated 264 C lymphoma with various concentrations of AZD, Ara-C, doxorubicin (DOX) singularly, or in combination. Our data showed that 264 C lymphoma cells underwent cell death upon single treatment of AZD, Ara-C, or DOX in a dose-dependent manner; furthermore, combined treatment of AZD/Ara-C or AZD/DOX enhanced cell death in 264 C lymphoma (Fig. 7A, B, Supplementary Fig. 5). We suggest that TRAF2/3-deficient 264 C lymphoma may serve as a model for testing new IAP antagonists.

We next determined whether WT or TRAF3 mutant human lymphomas exhibited differential sensitivity to IAP antagonist and whether IAP antagonist potentiated chemo-induced apoptosis in TRAF3-mutant lymphomas. To do so, we employed CRISPR/Cas9 technique to delete *traf3* gene in Ly1 human B cell lymphoma and verified the absence of TRAF3 protein in the KO lymphoma clone (Ly1-57) (Supplementary Fig. 6). Both Ly1 parental and Ly1-57 (TRAF3-KO) lymphoma cells were insensitive to Ara-C treatment since Ara-C (100μg/ml) caused a minimal level of cell death (Supplementary Fig. 7A). In contrast, Ly1-57 (TRAF3-KO) clone was significantly more sensitive to AZD treatment compared to Ly1 parental lymphoma (Fig. 7C, Supplementary Fig. 7B). Furthermore, AZD treatment sensitized resistant TRAF3-KO Ly1-57 lymphoma to Ara-C-induced cell death (Fig. 7C, Supplementary Fig. 7B). We also tested another TRAF3-KO subclone (Ly1-67) treated with Ara-C and/or AZD and found similar phenotypes to Ly1-57 (TRAF3-KO) (data not shown). We next tested another chemotherapeutic drug, DOX, routinely used to treat human B cell lymphomas. TRAF3-KO Ly1-57 lymphomas were more sensitive to DOX treatment compared to TRAF3-WT Ly1 lymphomas (Supplementary Fig. 7C). Importantly, TRAF3-KO Ly1-57 lymphoma was significantly more sensitive to AZD treatment; moreover, combined treatment of AZD and DOX caused a significantly higher level of cell death in TRAF3-KO Ly1-57 lymphoma than its WT counterpart (Fig. 7D, Supplementary Fig. 7D). Control without AZD treatment was shown in Supplementary Fig. 7A, C.

We also tested another human B cell lymphoma, Ly7, which has TRAF3 loss (Supplementary Fig. 6). Ara-C treatment (100 μg/ml) caused a low level of cell death in Ly7 (TRAF3-loss) lymphoma (∼8%), albeit higher than in Ly1 (TRAF3-WT) lymphoma (Supplementary Fig. 7E). In contrast, Ly7 lymphoma was extremely sensitive to AZD treatment (Fig. 7E, Supplementary Fig. 7F). DOX treatment led to a similar level of cell death in Ly7 and Ly1 (Supplementary Fig. 7G), while Ly7 lymphoma was still extremely sensitive to AZD treatment (Fig. 7F, Supplementary Fig. 7H). Combined treatment of Ara-C/AZD or DOX/AZD did not result in a higher level of cell death in Ly7 lymphoma compared to AZD single treatment (Fig. 7E, F, Supplementary Fig. 7F, H). Control without AZD treatment was shown in Supplementary Fig. 7E, G. Overall, we conclude that TRAF3 deficiency rendered lymphoma cells more sensitive to IAP antagonist treatment.

To further test whether TRAF2 deficiency or TRAF2/3 double deficiency also render lymphoma cells more sensitive to IAP antagonist treatment, we generated TRAF2-KO Ly1 cells (Ly1-P30) and TRAF2/3-DKO Ly1 cells (Ly1-K77) (Supplementary Fig. 8A). We treated Ly1 parental (Ly1-P), TRAF2-KO, TRAF3-KO, and TRAF2/3-DKO lymphoma cells with DOX, AZD, or AZD plus DOX (Supplementary Fig. 8B–E). Our data showed that TRAF2-KO, TRAF3-KO, and TRAF2/3-DKO lymphoma cells were more sensitive to AZD treatment alone or to combined treatment of AZD/DOX (Supplementary Fig. 8D, E). Taken together, we suggest that IAP antagonist plus chemotherapeutics may be more effective in treating TRAF2 or TRAF3 deficient B cell lymphomas.

## Discussion

We identified a new mechanism protecting B cells with single or double deficiency of TRAF2/3 against DNA damage-induced apoptosis, which may have important implications for B cell lymphomagenesis. We found that: (1) B-TRAF2-KO, B-TRAF3-KO and B-TRAF2/3-DKO B cells resisted to DNA damage-induced apoptosis; (2) B-TRAF3-KO and B-TRAF2/3-DKO B cells upregulated cIAP2 and XIAP, which in turn attenuated caspase-3 activation; (3) resistance to DNA damaged-induced apoptosis depended on NF-κB2 which is required for XIAP and cIAP2 transcription; (3) B-cell specific TRAF2/3 double deficiency resulted in B-cell lymphomagenesis with a relatively low frequency; (4) Sequencing BCR repertoire uncovered abnormal oligoclonal or clonal expansion of diseased B-TRAF2/3-DKO B cells; (5) diseased aged B-TRAF2/3-DKO B cells but not young primary B cells harbored chromosomal alterations; (6) blocking IAP activity sensitizes chemo-resistant TRAF2-deficient, TRAF3-deficient or TRAF2/3 double deficient B-cell lymphomas to chemotherapeutic drugs. These results suggest that TRAF2/3 deficiency per se does not cause B-cell malignancy frequently instead it permits B cells to survive better upon DNA damage to accumulate genetic alterations which may subsequently cause B-cell lymphomas. Our studies provide insight into mechanisms modulating DNA damage-induced apoptosis and may help to develop more effective treatment for TRAF2 or TRAF3 mutant B-cell lymphomas using IAP antagonists.

B cells deficient in TRAF2 or TRAF3 or both exhibit survival advantage over corresponding control B cells in the absence of survival factors such as BAFF [22, 24]. It has been presumed that this survival advantage is due to the elevated active NF-κB2 in TRAF2/3-deficient B cells. However, elevated NF-κB2 did not provide survival advantage to T cells or macrophages in the absence of TRAF3 [28, 29]. Hence, it was suggested that TRAF3 deficiency might enhance cell survival via elevating active NF-κB2 in a cell-type dependent manner [28]. Further studies indicate that the survival advantage of TRAF3-deficient B cells may be attributed to glucose metabolism as well as enhanced expression of *c-myc* or *Mcl-1* [33, 34]. We also observed that B-TRAF2/3-DKO B cells survived better than LMC without requiring survival factors (e.g., IL-4 or BAFF) after 24 h of cell culture (data not shown). Importantly, B-TRAF2/3-DKO B cells were remarkably more resistant to DNA damage-induced apoptosis, probably by upregulating XIAP and cIAP2, because blocking XIAP and cIAP2 abrogated the enhanced survival of B-TRAF2/3-DKO B cells. Of note, TRAF2 or TRAF3 single deficiency also protected B cells against DNA-damage-induced apoptosis, and there was no statistical difference between B-TRAF3-KO and B-TRAF2/3-DKO B cells, suggesting that the phenotypes of DKO B cells are likely attributed to *Traf3* deletion. Consistently, we found that both B-TRAF2/3-DKO and B-TRAF3-KO B cells exhibited an increased level of XIAP and cIAP2 expression. We employed genetic models to definitively demonstrate that NF-κB2 is essential for upregulated XIAP and cIAP2 transcription and enhanced survival in TRAF3-deficient B cells upon Ara-C treatment. Taken together, our study reveals a new cascade of NF-κB2/XIAP/cIAP2 pathway inhibiting apoptosis by converging at suppressing caspase-3 activation.

Chromosomal translocations are often associated with human B cell lymphomas and detected in mouse B cell lymphomas [31, 32, 35]. Since we did not detect an appreciable level of chromosomal alterations in primary B cells of young B-TRAF2/3-DKO mice, it suggests that TRAF2/3 deficiency per se does not lead to a high level of genomic instability in B cells; rather it permits a fraction of B cells to accumulate genetic alternations gradually. It is also possible that the frequency of translocations in primary B cells is below the detection threshold of SKY. Nevertheless, we observed recurrent chromosomal translocations in the diseased B cells from aged B-TRAF2/3-DKO mice. All the translocations involved chromosome 12 and 2, and the breakpoints were at the telomeric region of chromosome 12 where *Igh* locus is located. However, we failed to detect such translocations using whole-genome sequencing, likely due to the relatively low frequency of such events (only present in 20–30% of tumor population). Nevertheless, chromosomal translocations are not required for lymphoma development in B-TRAF2/3-DKO mice since 264 C lymphoma had normal karyotyping and lacked any detectable translocation. Of note, it is possible that 264 C lymphoma did harbor a low frequency of chromosomal translocations or other genetic alterations that need more sensitive methodology to detect. Overall, we suggest that deficiency in TRAF2 or TRAF3 may confer survival advantage to B cells via elevated NF-κB2 that predisposes B cells to lymphomagenesis by permitting them to accumulate low frequency genetic alterations, other than chromosomal translocations, that eventually cause lymphoma.

Chemo-resistance is one of the main causes for cancer-related death and remains a significant challenge of cancer treatment. IAPs may be promising targets that could counteract therapy resistance mechanisms and have been widely tested for treating different types of cancers [36–38]. The functions of IAP proteins are much more complicated than simply protecting cells against apoptosis through interacting with caspases. For instance, XIAP and cIAP2 can inhibit cell death execution by physically binding caspases or by mediating their proteasomal degradation. On the other hand, XIAP and cIAP2 can also trigger survival signaling by activating NF-κB pathways [39, 40]. Thus, perturbing the level of XIAP and cIAP2 protein may tip the delicate balance between NF-κB-driven survival vs. caspase-dependent apoptosis in cancer cells. In this regard, we found that human lymphoma cells that either contain TRAF3 loss (Ly-7) or have TRAF3 deleted (Ly-1-TRAF3-KO) were more sensitive to IAP antagonist treatment than their TRAF3 WT controls. Furthermore, IAP antagonist sensitized chemo-resistant lymphoma cells to apoptosis either alone or in combination with chemotherapeutic drugs (Ara-C or DOX). The question is why TRAF3-mutant lymphomas are more sensitive to IAP antagonist treatment. Our data showed that NF-κB2 can promote transcription of XIAP and cIAP2, while others have shown that XIAP and cIAP2 can activate NF-κB pathway [39, 40]. In the absence of TRAF3, this NF-κB2/XIAP/cIAP2 signaling cascade may constitute an autoregulatory positive feedback loop and TRAF3-mutant lymphoma cells may rely on this feedback mechanism for their survival. Alternatively, the level of XIAP and/or cIAP2 in mutant lymphomas may recalibrate the threshold of caspase activity that a cancer cell can withstand before committing to irreversible apoptosis cascade.

Our results suggest that sequencing BCR repertoire may serve as a useful approach to detect lymphoma cells in tumor tissue or peripheral blood mononuclear cells (PBMC). This method appears to be highly sensitive and be able to detect multiple expanded clones with accurate clonal frequency and unique CDR3 regions. PBMC samples are readily accessible and can be subject to BCR repertoire sequencing to detect different subclones of lymphoma cells that harbor the dominantly expanded CDR3 regions. Furthermore, comparing the BCR repertoires of tumor tissue vs. PBMC samples may allow the monitoring of clonal dynamics of lymphoma cells in a much more accessible manner and offer novel insight for developing new treatment strategies.

About 62.5% of B-TRAF3-KO mice developed lymphomas [23], although it has not been reported whether B-TRAF2-KO mice develop B-cell lymphomas. The incidence of lymphoma development in B-TRAF2/3-DKO mice is apparently lower than what reported in B-TRAF3-KO mice, an observation might be explained as follows: (1) The criteria to define B cell lymphomas may be different between current study and previous reports; (2) Environmental factors (e.g., microbiome) may affect B cell homeostasis and oncogenesis in B-TRAF3-KO or B-TRAF2/3-DKO mice since studies were carried out in different animal facilities, which may warrant future studies.

### Reporting summary

Further information on research design is available in the Nature Research Reporting Summary linked to this article.

## Supplementary information

Reporting Summary

Supplemental Figure 1-8

Supplemental Tables

Original Data File

## Data Availability

Data and materials are available upon request.
