# Supplementary material for: TRAF2/3 deficient B cells resist DNA damage-induced apoptosis via NF-κB2/XIAP/cIAP2 axis and IAP antagonist sensitizes mutant lymphomas to chemotherapeutic drugs
Source: Cell Death Dis. 2023 Sep 8;14(9):599. doi: 10.1038/s41419-023-06122-2 (PMC10485046; doi:10.1038/s41419-023-06122-2)
Supplement: Supplementary file 2 — Supplemental Figure 1-8 [file 41419_2023_6122_MOESM2_ESM.pdf]

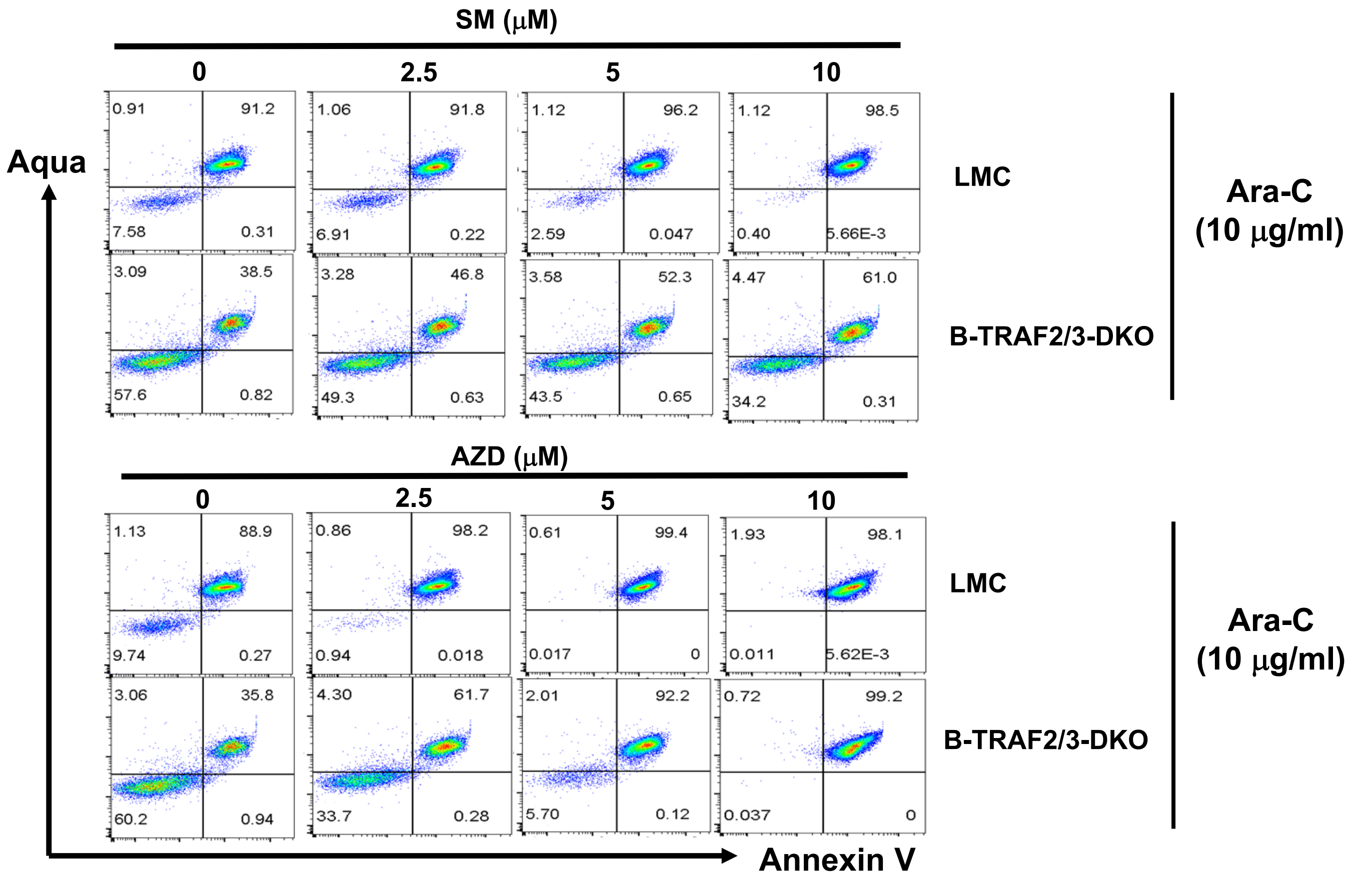

Supplemental Figure 1. B-TRAF2/3-DKO B cells survive better upon DNA damage in a XIAP and cIAP2 dependent manner. Representative flow cytometry data. LMC or B-TRAF2/3-DKO B cells cultured for 16 hours in the presence of Ara-C (10μg/ml) with indicated concentrations of a pan IAP inhibitor AZD5582 (AZD) or XIAP selective inhibitor SM-164 (SM). Cells were stained by Aqua (indicating dead cells) and Annexin-V (indicating apoptotic cells). Data were acquired and analyzed as described in methods. All experiments were independently repeated 3 or 4 times and each condition was performed in duplicates.

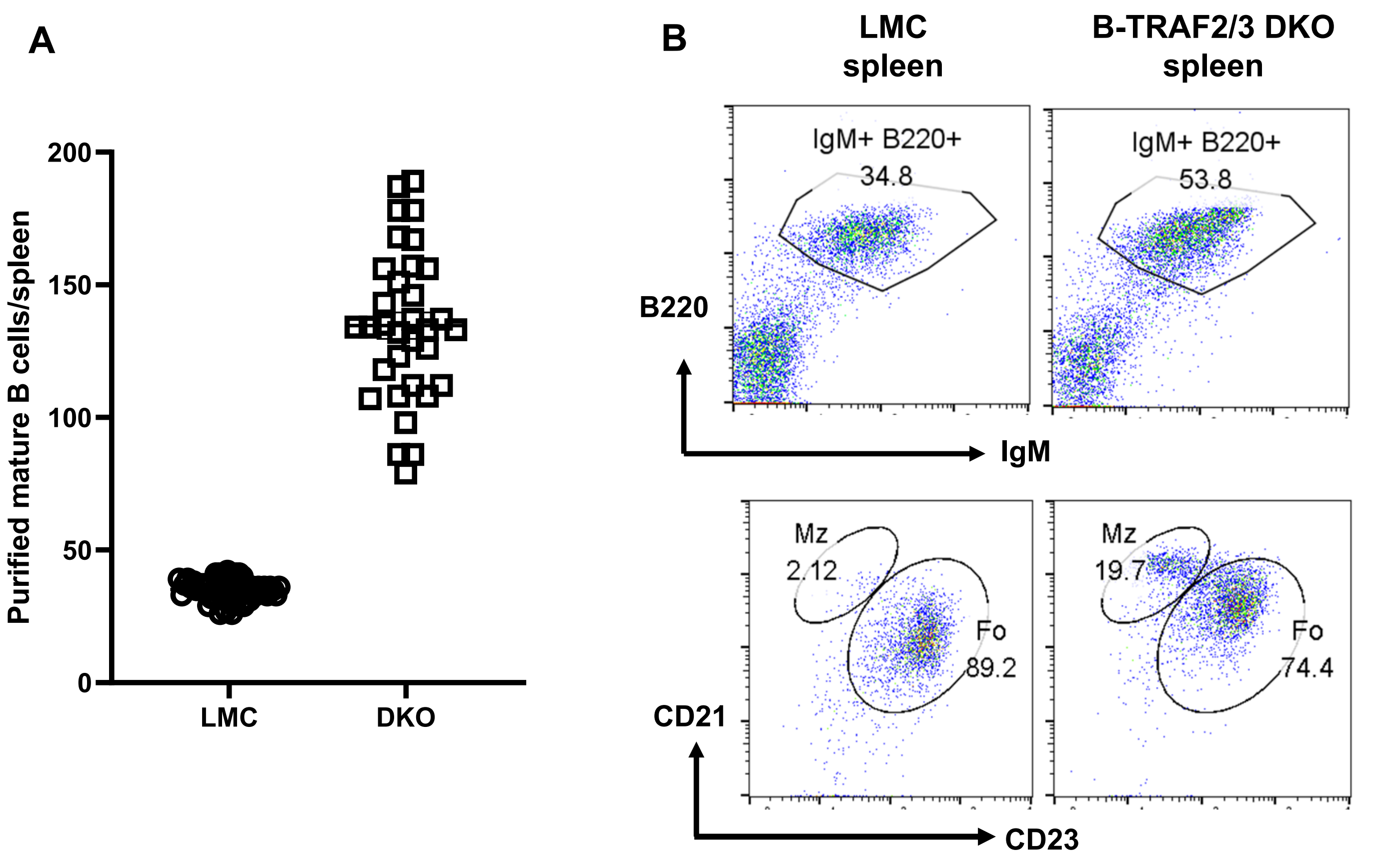

Supplementary Figure 2. Expansion of splenic B cells in B-TRAF2/3-DKO mice. (**A)** Summary of purified B cell numbers per mouse spleen. LMC: littermate control (n=60), DKO: B-TRAF2/3 double knockout (n=33). **(B)** Representative flow cytometry data of splenocytes of LMC (n>10) and B-TRAF2/3-DKO mice (n>10). Upper panel: B cell population (B220+/IgM+). Lower: Marginal zone (MZ) and Follicular (FO) B cells.

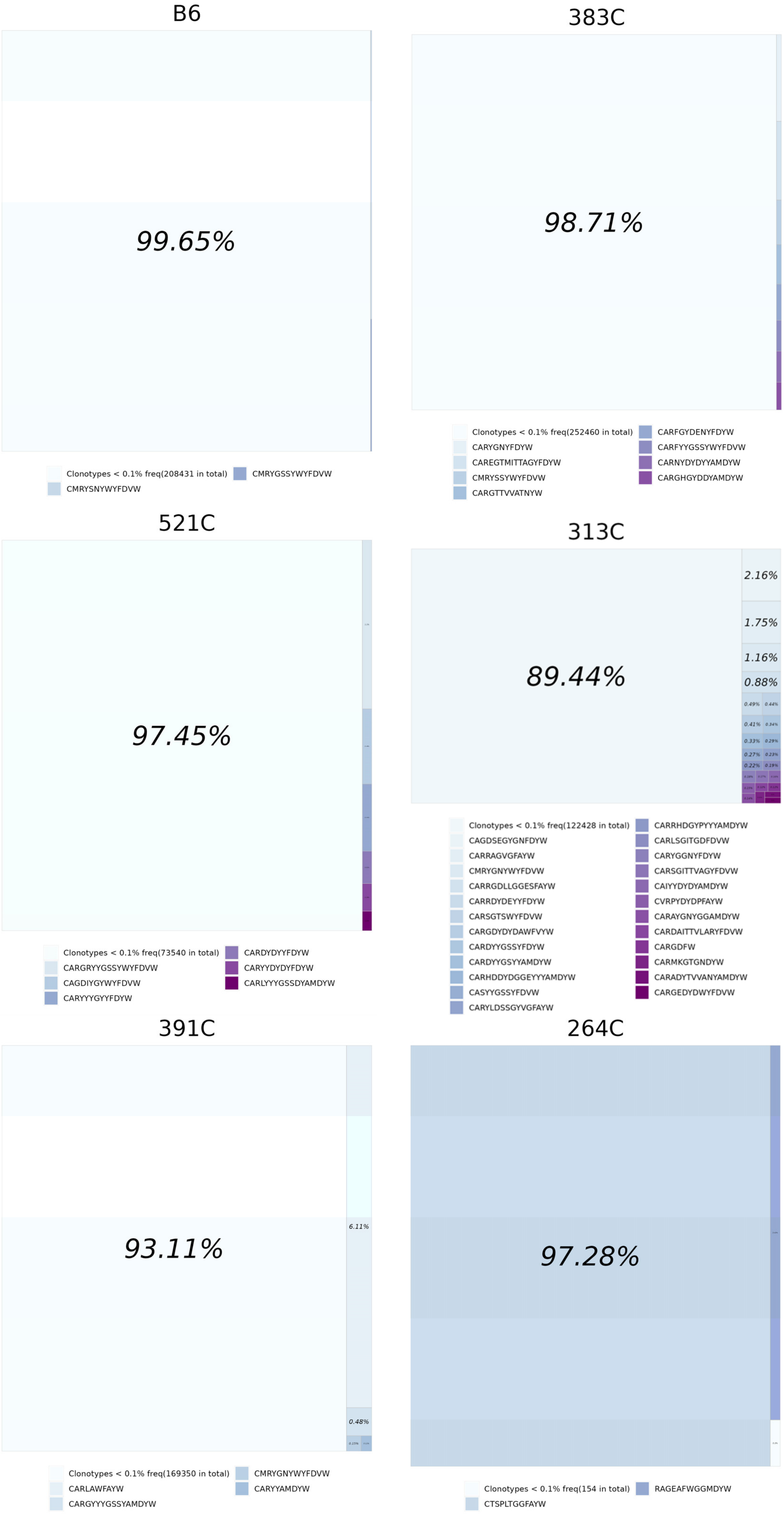

Supplemental Figure 3

Supplemental Figure 3. Abnormal oligo clonal and clonal expansion of B cells in diseased B-TRAF2/3-DKO mice. Square plots showing clonal frequency of different BCR clonotypes. Mice ID is indicated for each sample sequenced. Clonal frequency is calculated as the percentage of a given clonotype in an entire *Igh* repertoire of each sample sequenced. Clonotypes with low frequency (<0.1%) are pooled together and shown as one square, the most dominant one in all samples except in 264C. The most dominant clone in 264C is CTSPLTGGFAYW (97.28%), which is the lymphoma clone. The CDR3 a.a. sequences of expanded clonotypes (>0.1%) are shown below each square plot. The clonal frequency of each expanded clonotype is indicated in each square of the square plots.

264C

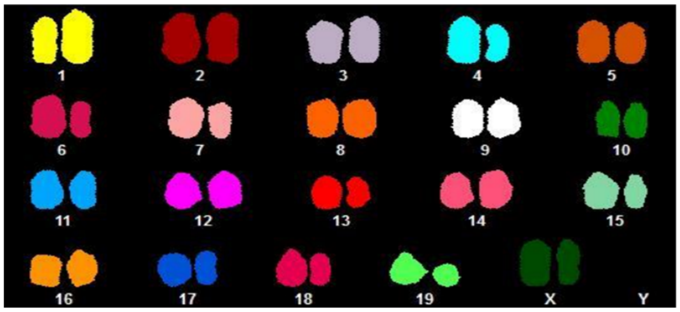

Supplemental Figure 4. Chromosomal alterations are not required for B cell lymphoma development in B-TRAF2/3-DKO mice. 264C B cells (0.5×10^6/ml, 6ml/well in 6-well plate) were cultured, treated with 100 ng/ml colcemid for 4 hour and metaphases spreads were prepared. SKY data showed no chromosomal alterations in 264C B cells. 12 metaphases were randomly chosen for full karyotype and analysis.

Supplemental Figure 4

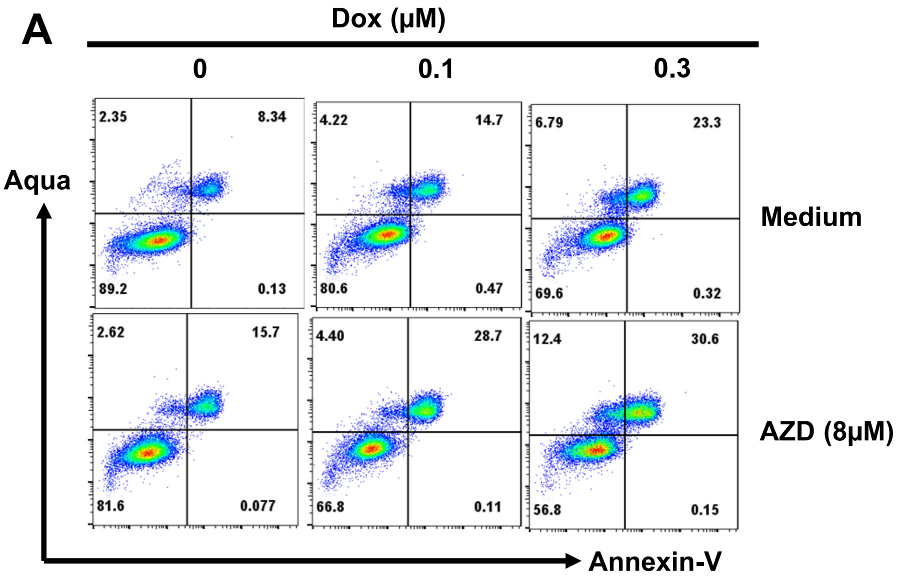

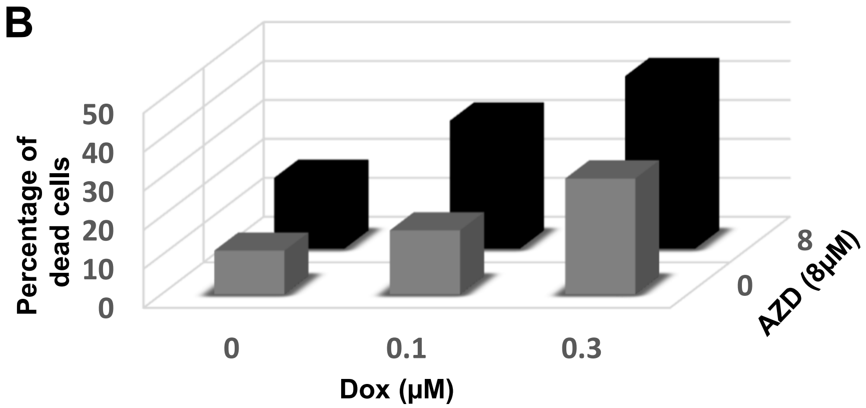

Supplemental Figure 5. Single treatment of DOX or AZD or combined treatment resulted in apoptosis in 264C lymphoma. (A) Representative flow cytometry data. Our newly established B-TRAF2/3-DKO B cell lymphoma line (264C) was cultured for 16 hours in the presence of indicated concentration of doxorubicin (Dox) with a pan IAP inhibitor AZD or without AZD (Medium). Cells were stained by Aqua (indicating dead cells) and Annexin-V (indicating apoptotic cells). Data were acquired and analyzed as described in methods. **(B)** Quantification of the percentage of dead cells from duplicated flow data shown in panel A. Experiments were independently repeated 3 times.

Supplemental Figure 5

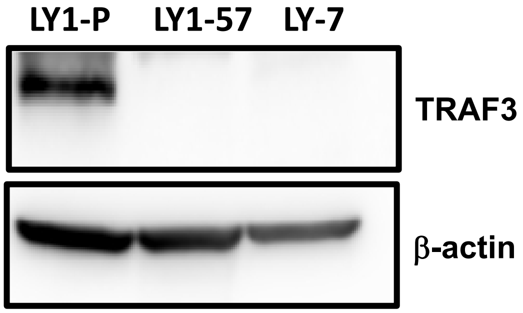

Supplemental Figure 6. The absence of TRAF3 protein expression in human B cell lymphoma lines. Representative western blotting data showing TRAF3 protein expression in different cell lines. LY1-P: LY1 parental cells that express TRAF3 (TRAF3-WT); LY1-57: LY1 clone whose TRAF3 gene was disrupted by gRNA/CRISPR/Cas9 (TRAF3-KO). LY-7: LY-7 parental line that has no detectable TRAF3 protein expression (TRAF3-loss). β-actin as protein loading control. Experiments were independently repeated 3 times.

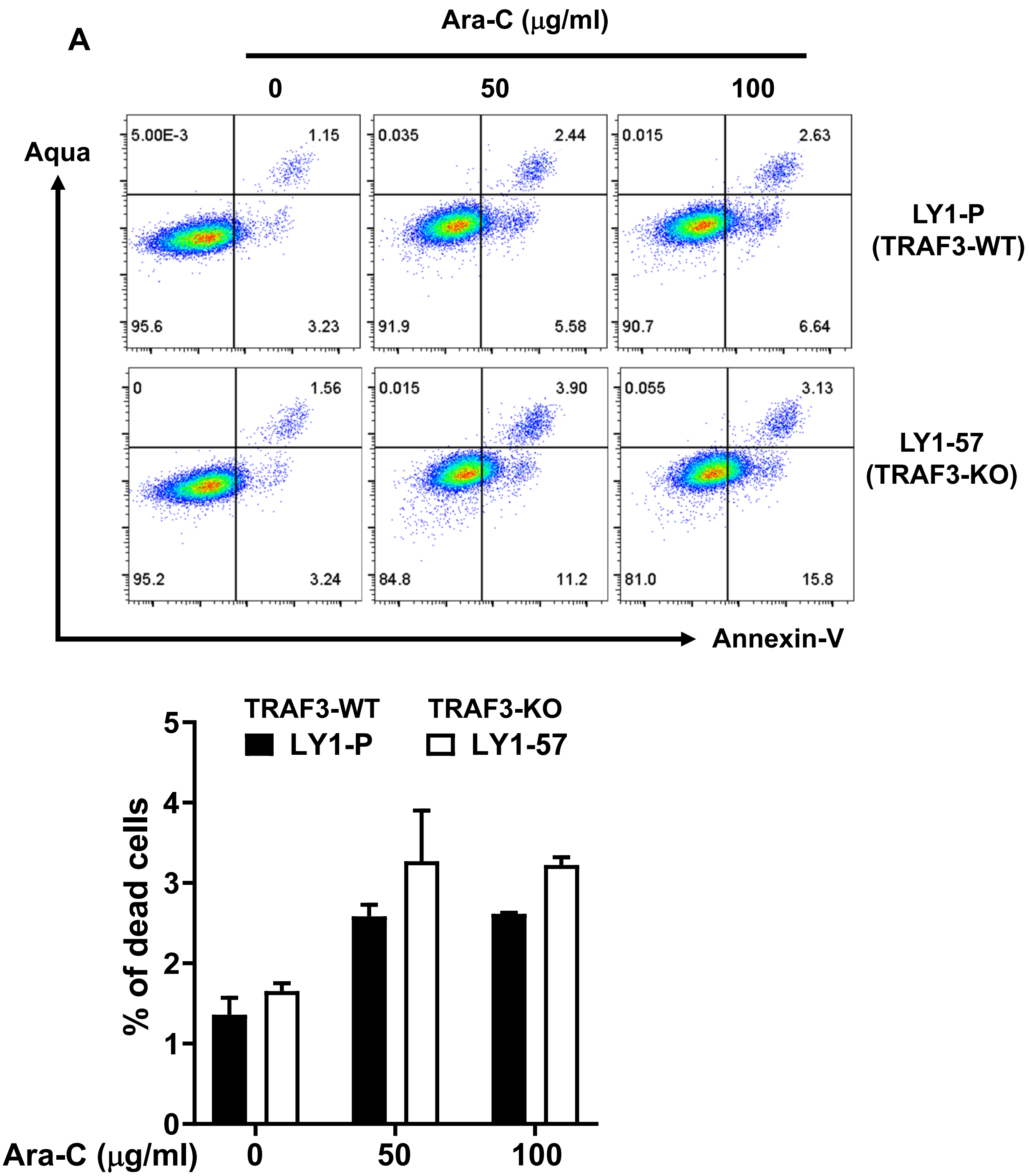

Supplementary Figure 7. TRAF3-deficient human B cell lymphomas are more sensitive to IAP antagonist treatment. (**A) (Upper)** Representative flow cytometry data showing insensitivity of human B cell lymphoma cell lines to Ara-C. Lymphoma cells were cultured for 16 hours in the presence of indicated concentration of Ara-C. Cells were stained by Aqua (indicating dead cells) and Annexin-V (indicating apoptotic cells). Data were acquired and analyzed as described in methods. **(Lower)** Quantification of the percentage of dead cells from duplicated flow data shown in upper panel. LY1-P: LY1 parental line that expresses TRAF3 (TRAF3-WT); LY1-57: LY1 clone whose TRAF3 gene was disrupted by gRNA/CRISPR/Cas9 (TRAF3-KO). Experiments were independently repeated 3 times.

Supplemental Figure 7A

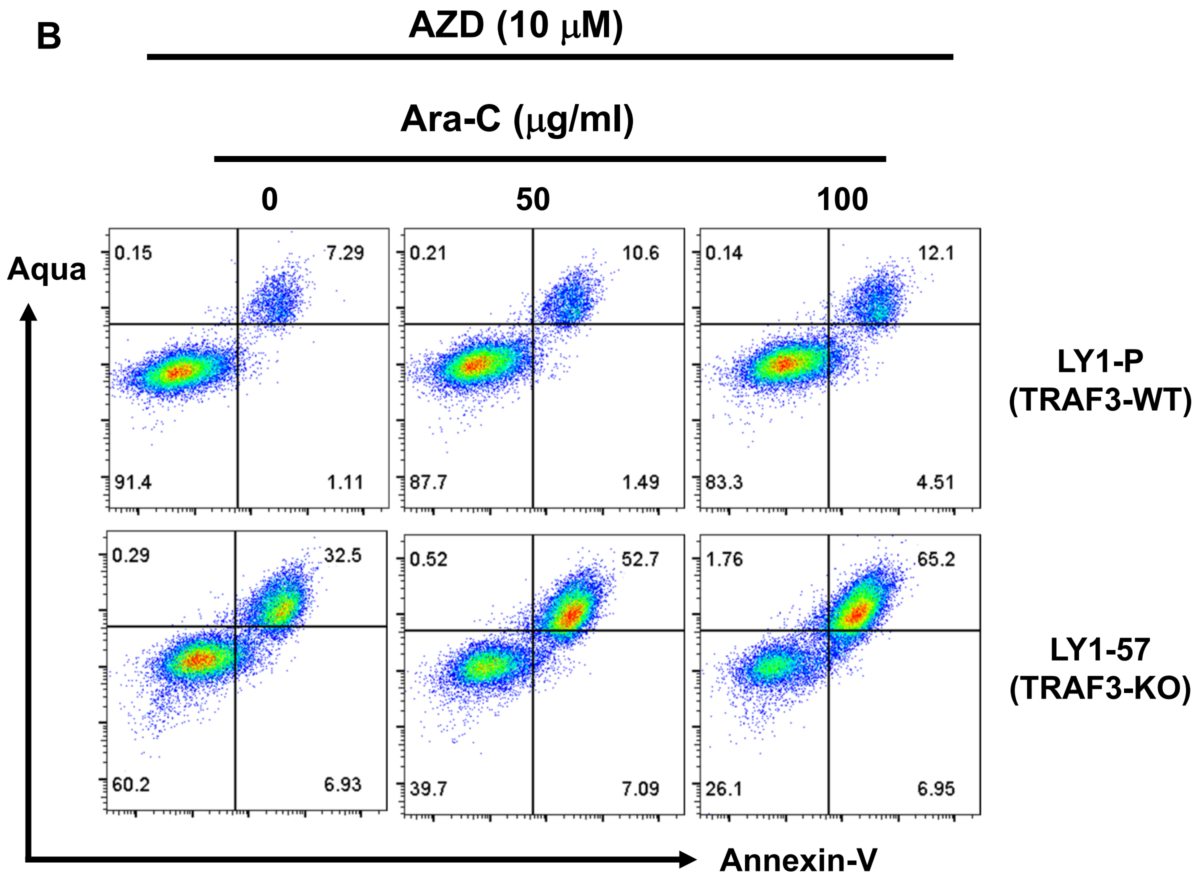

Supplementary Figure 7. TRAF3-deficient human B cell lymphomas are more sensitive to IAP antagonist treatment. (B) Representative flow cytometry data showing sensitivity of TRAF3-KO human B cell lymphoma lines to combined treatment of AZD and Ara-C. Experiments were performed as described in panel A. LY1-P: LY1 parental line that expresses TRAF3 (TRAF3-WT); LY1-57: LY1 clone whose TRAF3 gene was disrupted by gRNA/CRISPR/Cas9 (TRAF3-KO). Experiments were independently repeated 3 times.

Supplemental Figure 7B

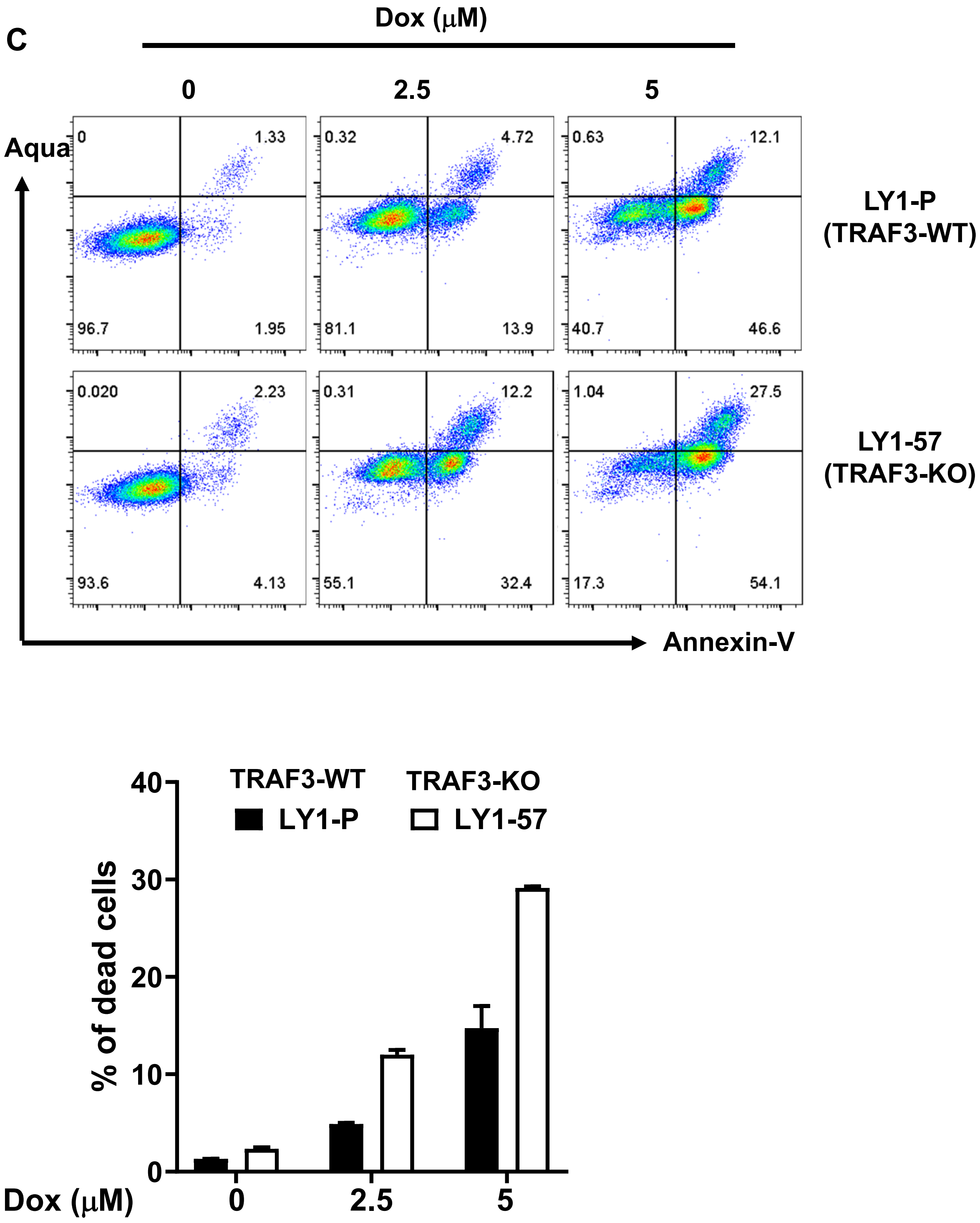

Supplementary Figure 7. TRAF3-deficient human B cell lymphomas are more sensitive to IAP antagonist treatment. (C) (Upper) Representative flow cytometry data showing sensitivity of human B cell lymphoma cell lines to doxorubicin (Dox). Experiments were performed as described in panel A. **(Lower)** Quantification of the percentage of dead cells from duplicated flow data shown in Upper panel. LY1-P: LY1 parental line that expresses TRAF3 (TRAF3-WT); LY1-57: LY1 clone whose TRAF3 gene was disrupted by gRNA/CRISPR/Cas9 (TRAF3-KO). Experiments were independently repeated 3 times.

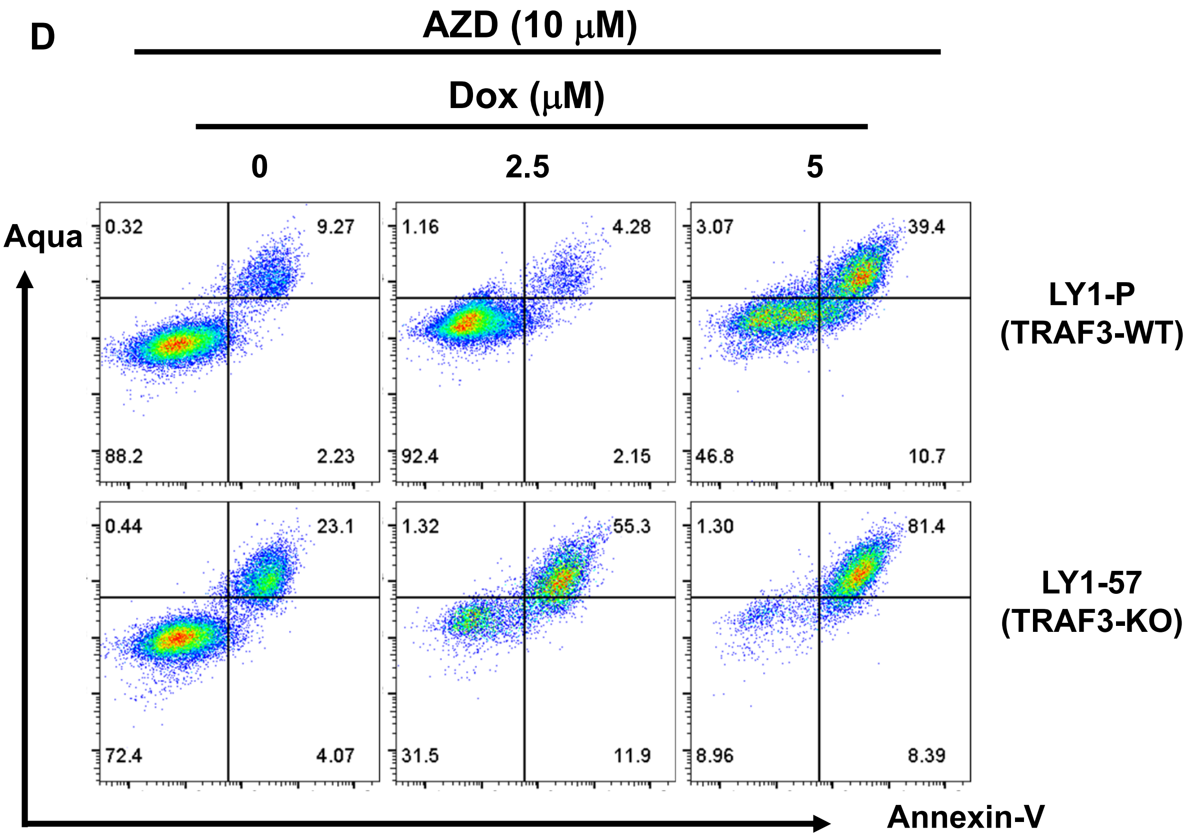

Supplementary Figure 7. TRAF3-deficient human B cell lymphomas are more sensitive to IAP antagonist treatment. (D) Representative flow cytometry data showing sensitivity of TRAF3-KO human B cell lymphoma to combined treatment of AZD and Dox. Experiments were performed as described in panel A. LY1-P: LY1 parental line that expresses TRAF3 (TRAF3-WT); LY1-57: LY1 clone whose TRAF3 gene was disrupted by gRNA/CRISPR/Cas9 (TRAF3-KO). Experiments were independently repeated 3 times.

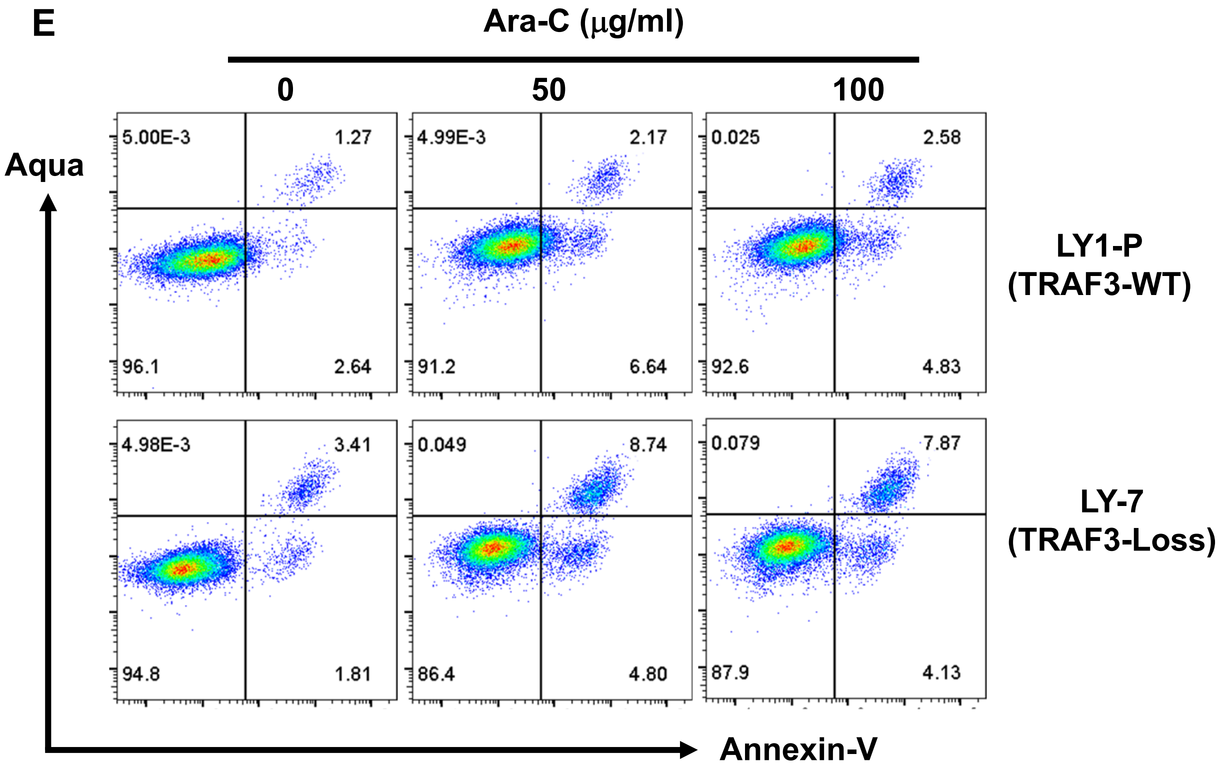

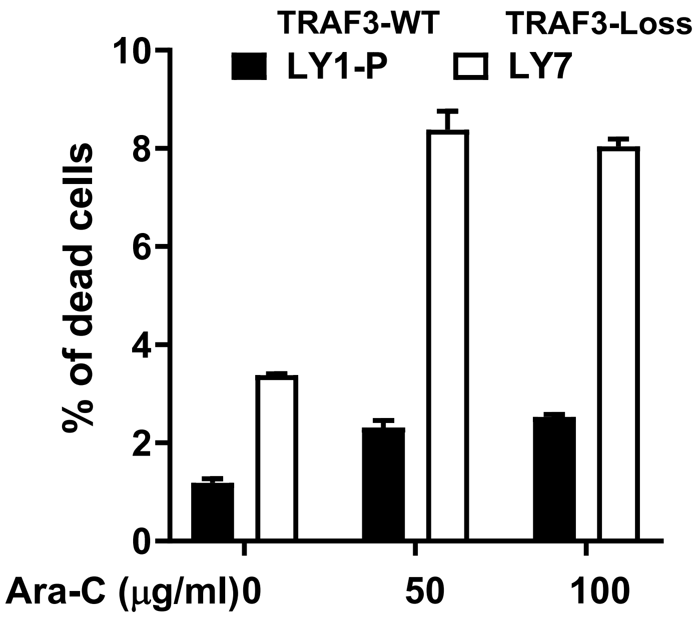

Supplementary Figure 7. TRAF3-deficient human B cell lymphomas are more sensitive to IAP antagonist treatment. (E) (Upper). Representative flow cytometry data showing sensitivity of TRAF3-loss human B cell lymphoma to Ara-C. Experiments were performed as described in panel A. **(Lower)** Quantification of the percentage of dead cells from duplicated flow data shown in Upper panel. LY1-P: LY-1 parental line that expresses TRAF3 (TRAF3-WT); LY-7: LY-7 parental line that has no detectable TRAF3 protein expression (TRAF3-loss). Experiments were independently repeated 3 times.

Supplemental Figure 7E

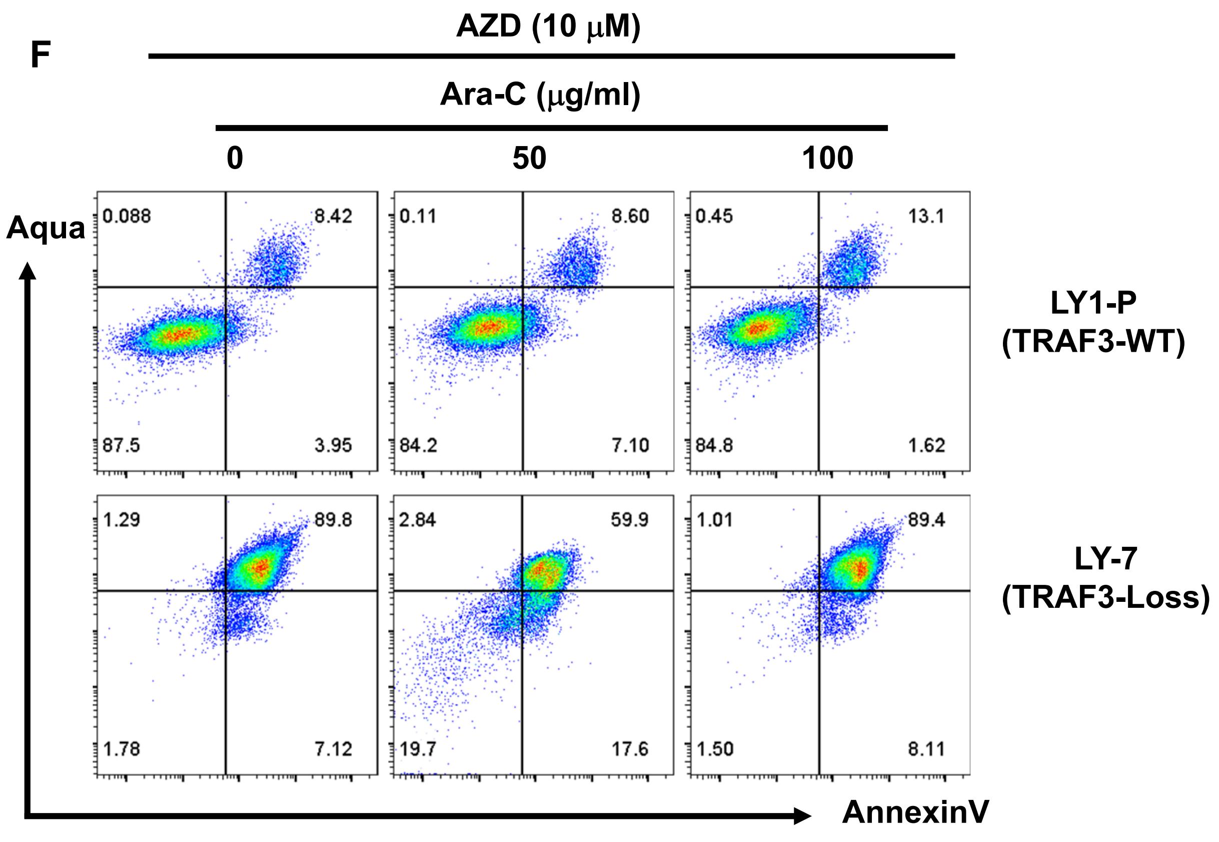

Supplementary Figure 7 TRAF3-deficient human B cell lymphomas are more sensitive to IAP antagonist treatment. (F) Representative flow cytometry data showing increased sensitivity of TRAF3-loss human B cell lymphoma to combined treatment of AZD and Ara-C. Experiments were performed as described in panel A. LY1-P: LY-1 parental line that expresses TRAF3 (TRAF3-WT); LY-7: LY-7 parental line that has no detectable TRAF3 protein expression (TRAF3-loss). Experiments were independently repeated 3 times.

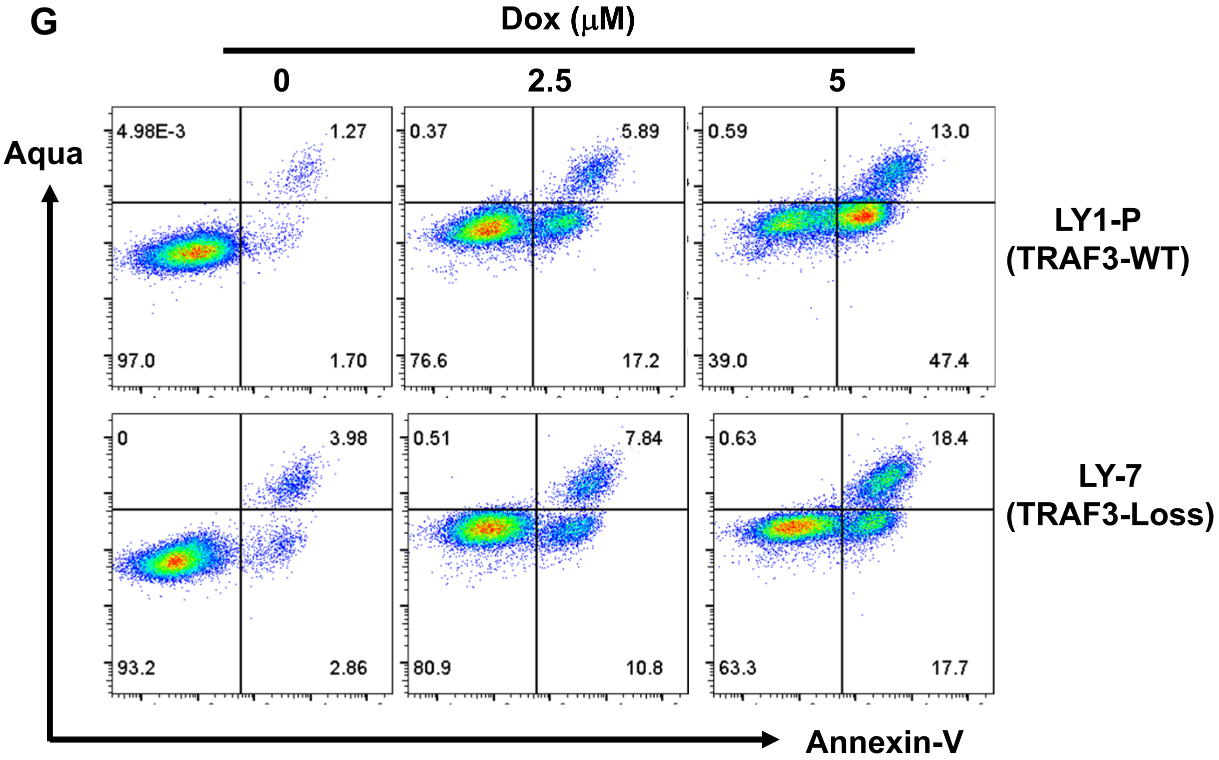

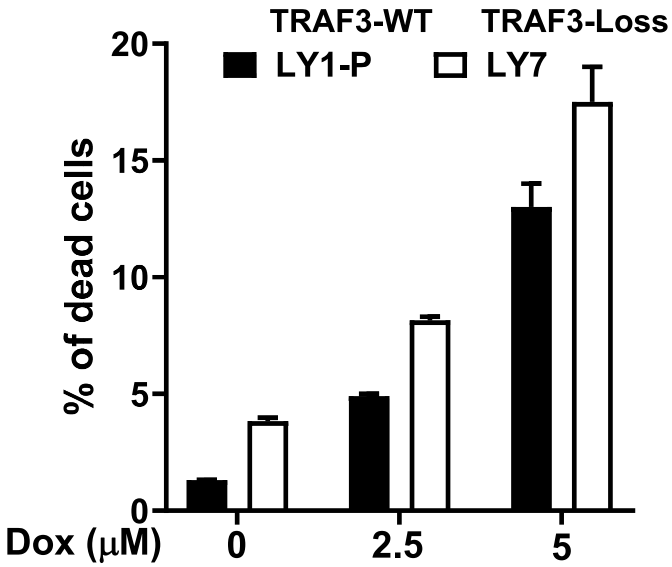

Supplementary Figure 7. TRAF3-deficient human B cell lymphomas are more sensitive to IAP antagonist treatment. (G) (Upper) Representative flow cytometry data showing sensitivity of human B cell lymphoma lines to doxorubicin (Dox). Experiments were performed similarly as described in panel A. **(Lower)** Quantification of the percentage of dead cells from duplicated flow data shown in Upper panel. LY1-P: LY1 parental line that expresses TRAF3 (TRAF3-WT); LY-7: LY-7 parental line that has no detectable TRAF3 protein expression (TRAF3-loss). Experiments were independently repeated 3 times.

Supplemental Figure 7G

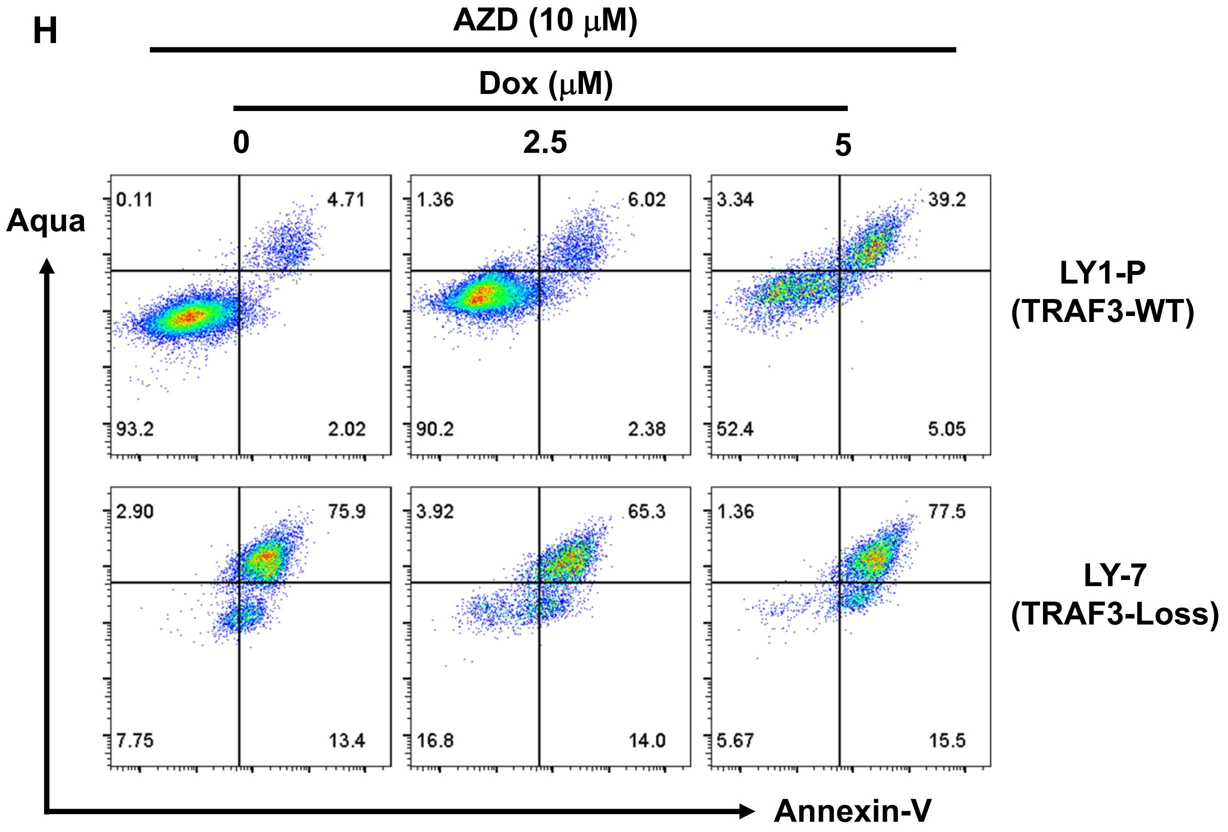

Supplementary Figure 7. TRAF3-deficient human B cell lymphomas are more sensitive to IAP antagonist treatment. (H) Representative of flow cytometry data showing increased sensitivity of TRAF3-loss human B cell lymphoma line to combined treatment of AZD and Dox. Experiments were performed similarly as described in panel A. LY1-P: LY1 parental line that expresses TRAF3 (TRAF3-WT); LY-7: LY-7 parental line that has no detectable TRAF3 protein expression (TRAF3-loss). Experiments were independently repeated 3 times.

Supplemental Figure 7H

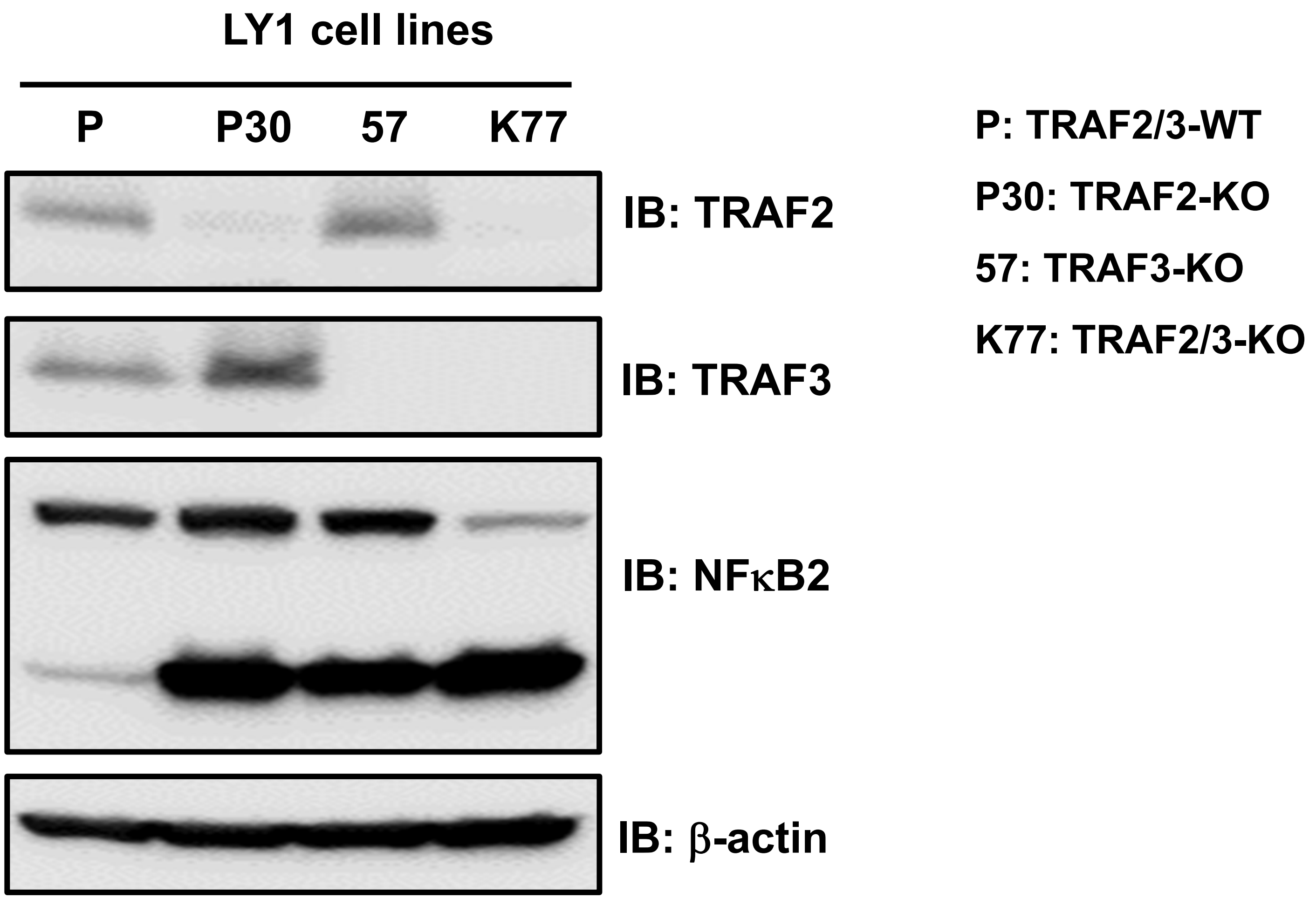

Supplemental Figure 8A. Establishment and confirmation of TRAF2 or TRAF3 and double deficient LY1 human B cell lymphoma lines. TRAF2 and/or TRAF3 was deleted in LY1 cells via CRIPSR/Cas9 approach (see Methods). TRAF2 and/or TRAF3 deletion clones were confirmed by western blot. Panel A is a representative western blotting data showing TRAF2, TRAF3, NF-κB2 protein expression in different cell lines. LY1-P: LY1 wild type or parental cells that express TRAF2 and TRAF3 (TRAF3-WT); LY1-P30: LY1 clone whose TRAF2 gene was disrupted by gRNA/CRISPR/Cas9 (TRAF2-KO); LY1-57: LY1 clone whose TRAF3 gene was disrupted by gRNA/CRISPR/Cas9 (TRAF3-KO); LY1-K77: LY1 clone whose TRAF2 and TRAF3 genes were disrupted by gRNA/CRISPR/Cas9 (TRAF2/3-DKO). β-actin as protein loading control.

Supplemental Figure 8A

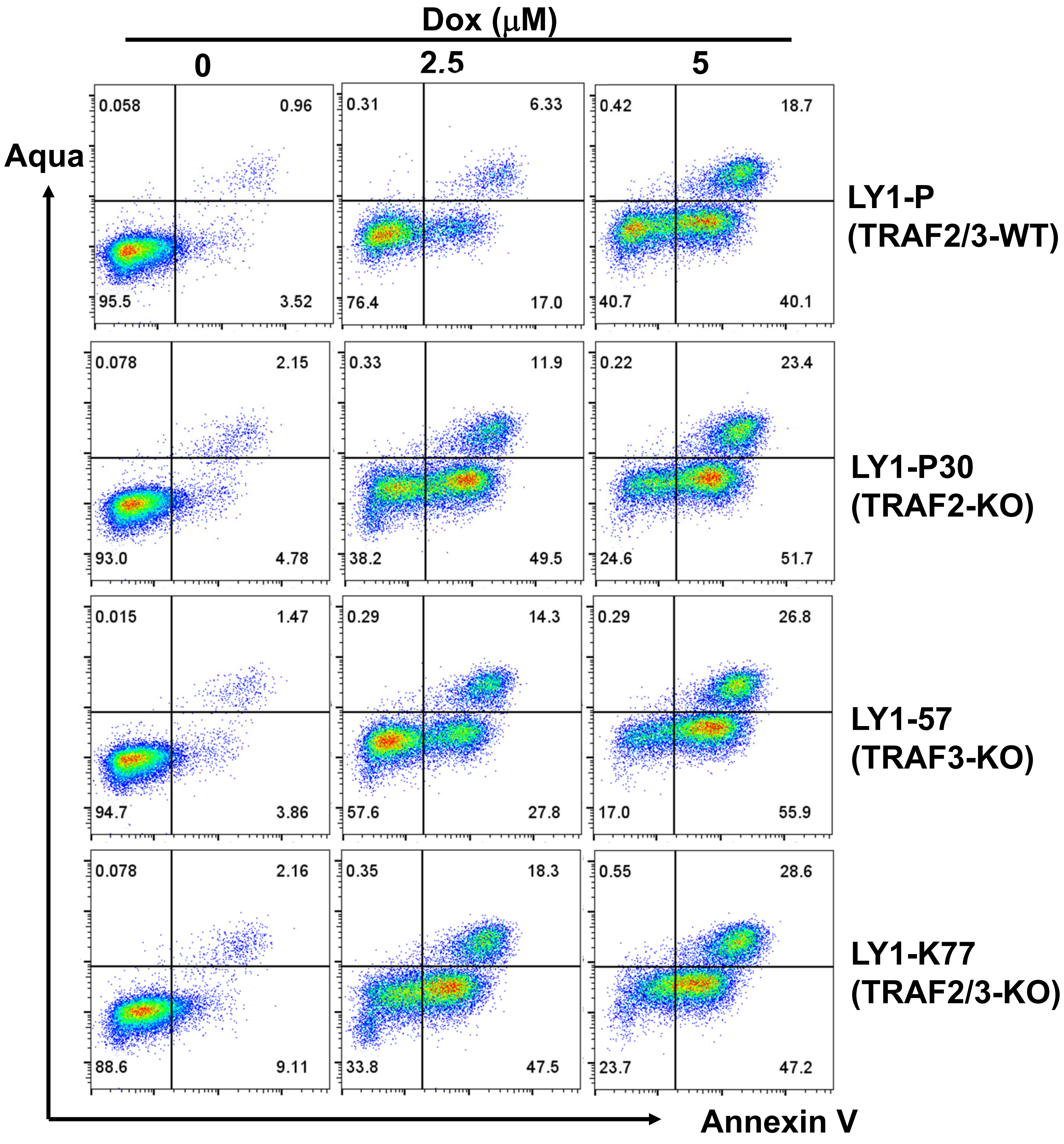

Supplemental Figure 8B

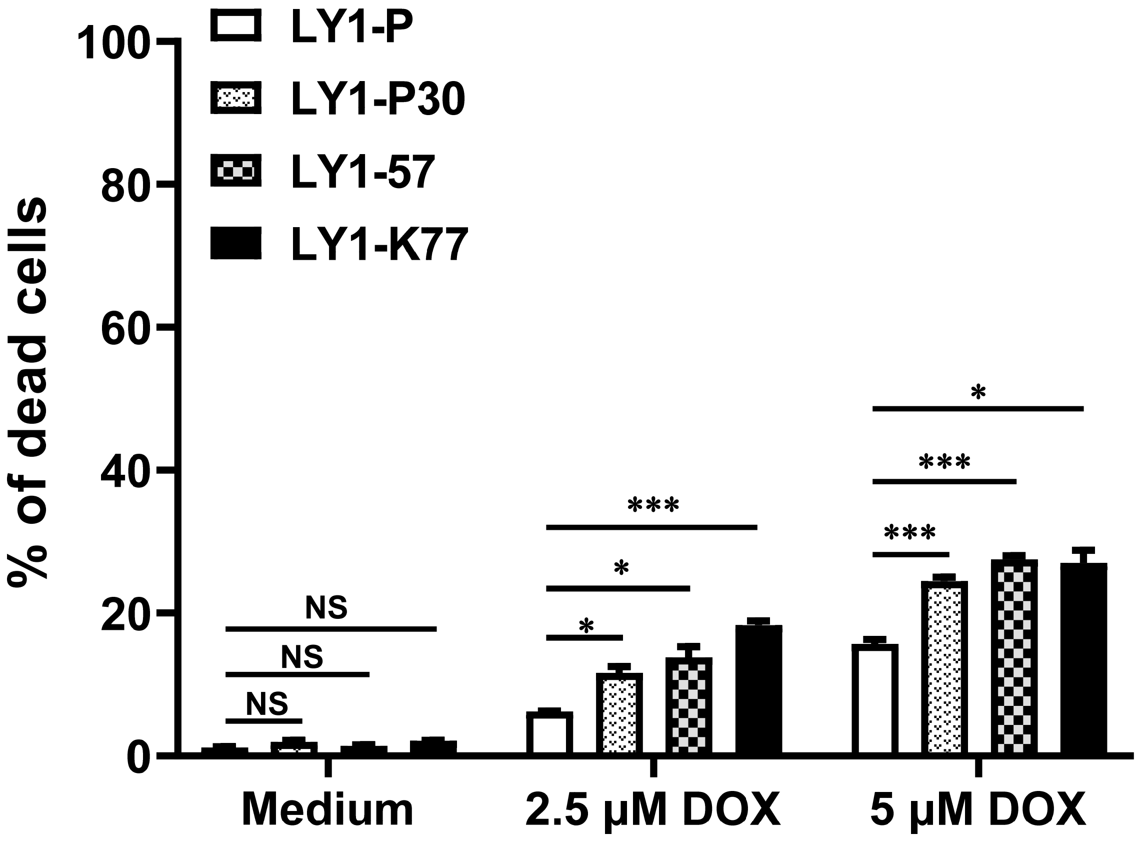

Supplemental Figure 8C

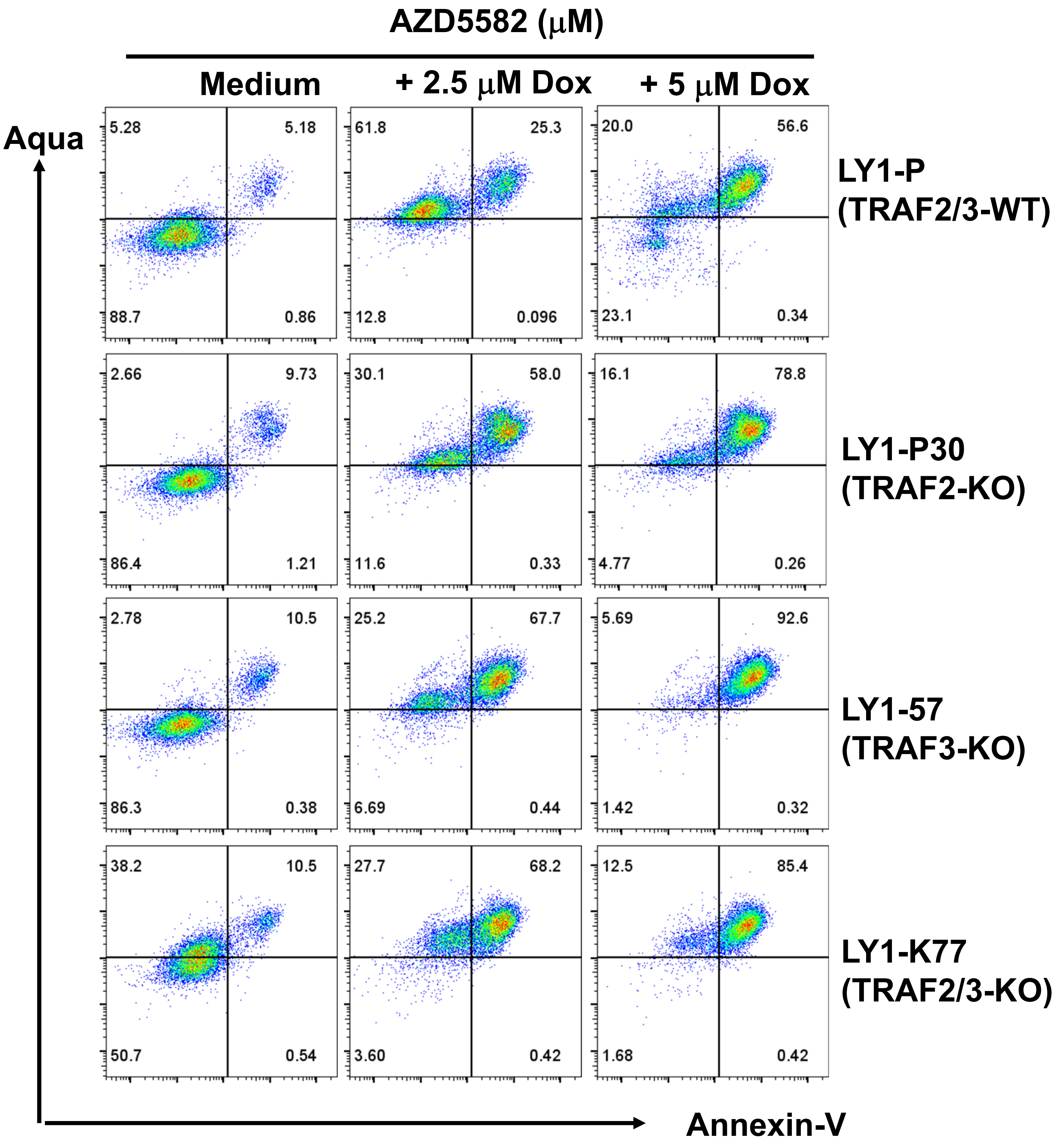

Supplemental Figure 8D

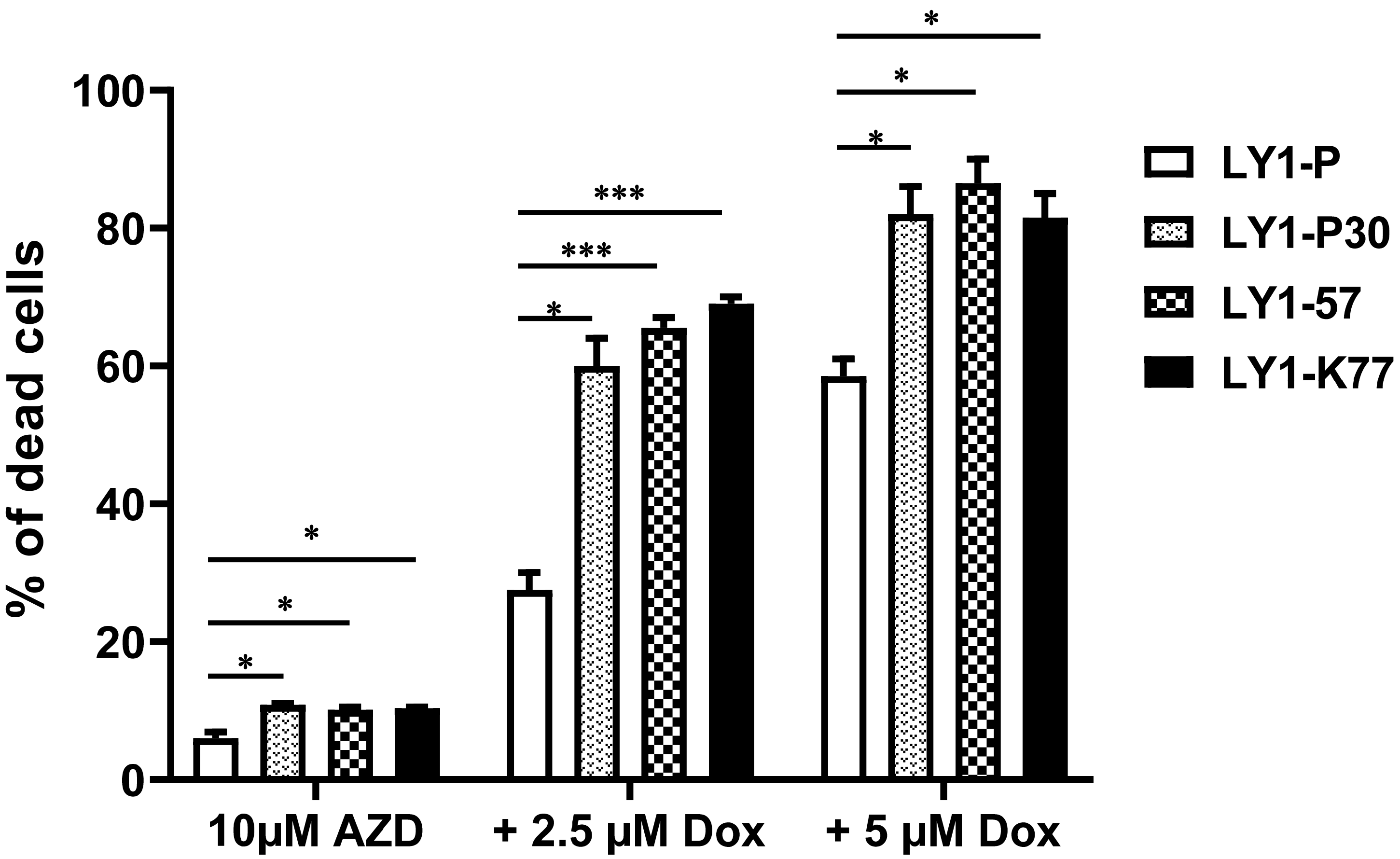

Supplementary Figure 8B-E. TRAF2 and/or TRAF3 deficiency sensitizes human B cell lymphomas to IAP antagonist and chemotherapeutic drug. LY1 parental cells (LY1-P), TRAF2-KO LY1 cells (LY1-P30), TRAF3-KO LY1 cells (LY-57) and TRAF2/3-DKO LY1 cells (LY1-K77) were cultured with different concentrations of doxorubicin (Dox) for 16 hrs in panel **B** and **C** or pretreated with 10 µM AZD5582 (AZD) for 30 minutes, then cultured with various concentrations of Dox for 16 hrs in panel **D** and **E**. **(B and D)** Representative flow cytometry data. **(C and E)** Quantification of the dead cell percentages from duplicated flow data shown in panel B or D, respectively. All experiments were independently repeated 3 to 4 times. Statistical difference was calculated with multiple t test using Graphpad prism.

Supplemental Figure 8E
