# Supplementary material for: TRAF2/3 deficient B cells resist DNA damage-induced apoptosis via NF-κB2/XIAP/cIAP2 axis and IAP antagonist sensitizes mutant lymphomas to chemotherapeutic drugs
Source: Cell Death Dis. 2023 Sep 8;14(9):599. doi: 10.1038/s41419-023-06122-2 (PMC10485046; doi:10.1038/s41419-023-06122-2)
Supplement: Supplementary file 3 — Supplemental Tables [file 41419_2023_6122_MOESM3_ESM.pdf]

Supplemental Table 1. Antibodies used in the study

Antibody	Clone	Catalogue number	Company
**Western antibodies**			
**Anti-Caspase3**	Poly	9662S	Cell signaling Biotechnology
**Anti-Caspase9**	C9	9508S	Cell signaling Biotechnology
**Anti- XIAP**	Poly	A6869	ABclonal
**Anti-c-IAP2**	Ac-71	SC-517317	Santa Cruz Biotechnology
**Anti-Akt**	C67E7	C67E7	Cell signaling Biotechnology
**Anti-NF-k B2 p100/52**	Poly	4882S	Cell signaling Biotechnology
**Anti-TRAF3**	Poly	SC-1828(H122)	Santa Cruz Biotechnology
**Anti-TRAF2**	Poly	4712P	Cell signaling Biotechnology
**Anti-β-actin**	C4	SC-47778	Santa Cruz Biotechnology
**HRP-anti-mouse-IgG**	Poly	115-035-166	Jackson ImmunoResearch
**HRP-anti-rabbit-IgG**	Poly	711-035-152	Jackson ImmunoResearch
**Flow Antibodies**			
**FITC-anti-mouse B220**	RA36B2	11045286	ebiosciences
**APC/Cy7-anti-mouse IgM**	RMM-1	406515	Biolegend
**PE-anti-mouse CD21**	7G6	55295	BDBioscience
**Alexa 647-anti-mouse CD23**	B3B4	101612	Biolegend
**Alexa 647-anti-mouse γ-H2AX**	N1-431	560447	BD Pharmingen
**Alexa 647 anti-rabbit IgG**	Poly	111-605-144	Jackson ImmunoResearch

Supplemental Table 2. Real-time PCR and ChIP primers

**Real-time primers**			
**Gene**	**Primer Type**	**Primer sequences (5' to 3')**	**PCR Conditions**
**XIAP**	F<br>R	ACGGATTGGAAGCCAAGTGA<br>GCAGTTCTTCCCAAAGATTCCT	94°C 5 min, 95°C 20s, 60°C 15s, 72°C 15s, 30 cycles, 72°C 10 min
**cIAP2**	F<br>R	ATGCAGACGCAGCAAGTATGTA<br>AGCACATCAGCCTTCCACTTC	94°C 5 min, 95°C 20s, 60°C 15s, 72°C 15s, 30 cycles, 72°C 10 min
**Caspase9**	F<br>R	TGGATGCTGTGTCAAGTTTGC<br>CAACCCTGAGAAGGAGGGACT	94°C 5 min, 95°C 20s, 60°C 15s, 72°C 15s, 30 cycles, 72°C 10 min
**β-actin**	F<br>R	TGGAATCCTGTGGCATCCATGAAAC<br>TAAAACGCAGCTCAGTAACAGTCCG	94°C 5 min, 95°C 20s, 60°C 15s, 72°C 15s, 30 cycles, 72°C 10 min
**ChIP primers**			
**Gene**	**Primer Type**	**Primer sequences (5' to 3')**	**PCR Conditions**
**XIAP**	F<br>R	CTTGGTTCCGGTCTTCGAGTT<br>AGGAGAAGGAAACGGAAGTCAC	94°C 5 min, 95°C 20s, 60°C 15s, 72°C 15s, 30 cycles, 72°C 10 min
**cIAP2**	F<br>R	TTCAGTAAATGACACGAAGAG<br>ACCTGCGGTGCTTTCCT	94°C 5 min, 95°C 20s, 60°C 15s, 72°C 15s, 30 cycles, 72°C 10 min

Supplementary Table 3: Summary of diseased B-TRAF2/3-DKO mice

ID	Diagnosis	Life-span	B220	CD19	IgM	IgA	Continuous *in vitro* growth	*In vivo* growth
**93C**	splenomegaly	18 months	positive	positive	positive	negative	No	No
**166C**	splenomegaly	13 months	positive	positive	positive	negative	No	No
**226C**	splenomegaly	14 months	positive	positive	positive	negative	No	No
**237C**	splenomegaly	15 months	positive	positive	positive	negative	No	No
**264C**	splenomegaly	14 months	positive	positive	negative	positive	Growth	Growth
**313C**	splenomegaly	16 months	positive	positive	positive	negative	No	No
**315C**	splenomegaly	15 months	positive	positive	positive	negative	No	No
**329C**	ascites	12 months	positive	positive	positive	negative	No	No
**383C**	ascites	12 months	positive	positive	positive	negative	No	No
**391C**	ascites	13 months	positive	positive	positive	negative	No	No
**521C**	splenomegaly	11 months	positive	positive	positive	negative	No	No

Eleven out of 28 B-TRAF2/3-DKO mice developed splenomegaly or ascites. Different growth media were tested including lymphocyte medium or STEM cell medium.
