# Supplementary material for: TRAF2/3 deficient B cells resist DNA damage-induced apoptosis via NF-κB2/XIAP/cIAP2 axis and IAP antagonist sensitizes mutant lymphomas to chemotherapeutic drugs
Source: Cell Death Dis. 2023 Sep 8;14(9):599. doi: 10.1038/s41419-023-06122-2 (PMC10485046; doi:10.1038/s41419-023-06122-2)
Supplement: Supplementary file 4 — Original Data File [file 41419_2023_6122_MOESM4_ESM.pdf]

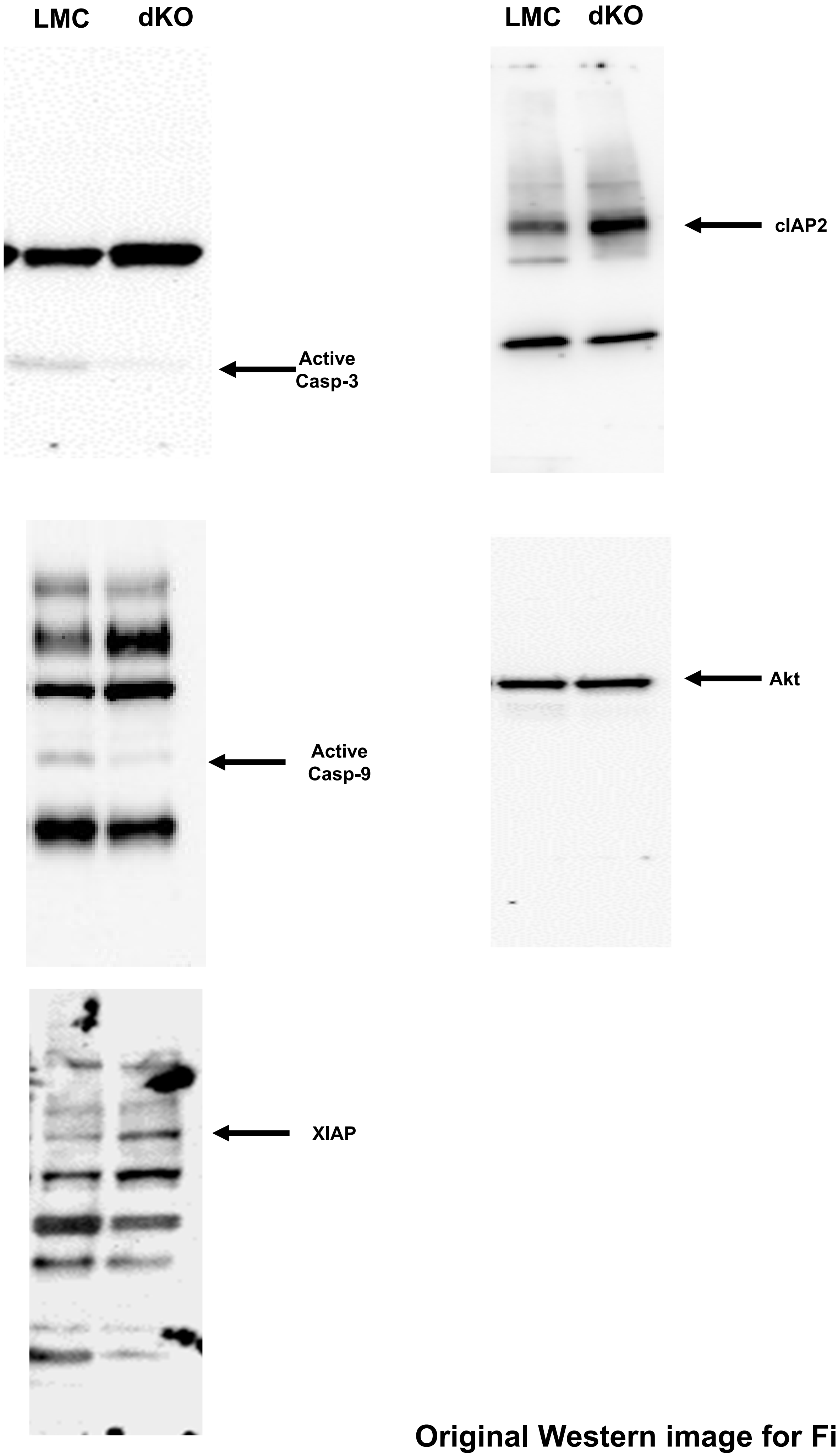

Original Western image for Figure 2C

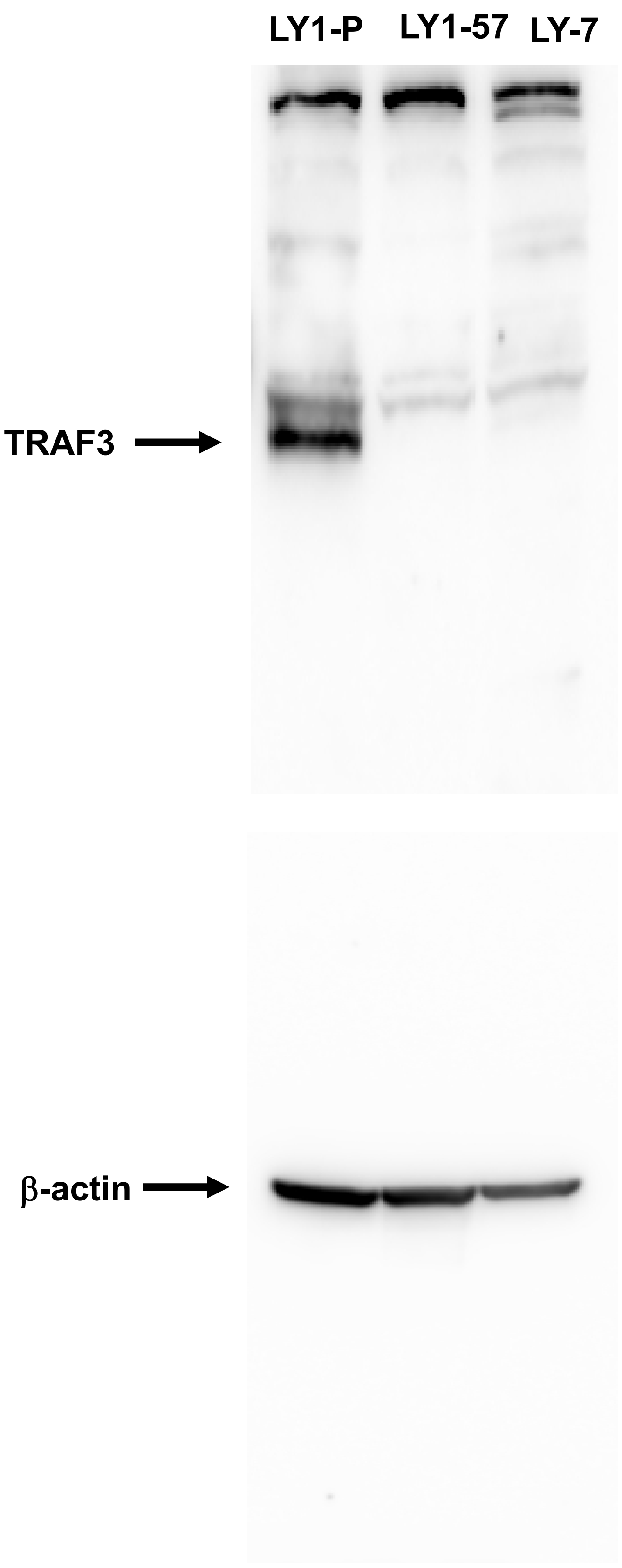

Original western images for Supplemental Figure 6

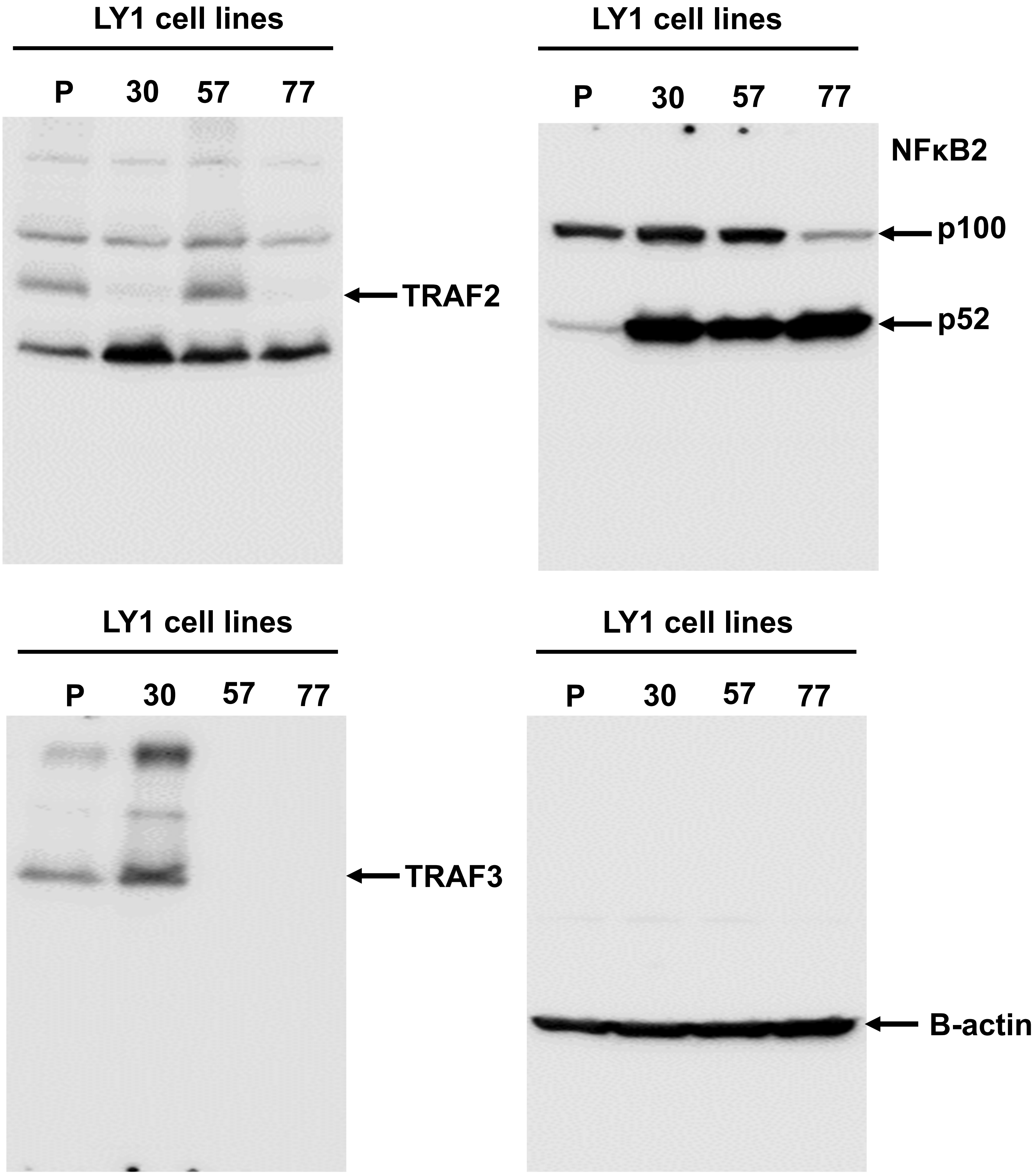

Original western images for Supplemental Figure 8A
